# Effect of a Patient Decision Aid on Preferences for Colorectal Cancer Screening Among Older Adults: A Secondary Analysis of a Randomized Clinical Trial

**DOI:** 10.1001/jamanetworkopen.2022.44982

**Published:** 2022-12-05

**Authors:** Alexandra F. Dalton, Carol E. Golin, Carolyn Morris, Christine E. Kistler, Rowena J. Dolor, Kaitlyn B. Bertin, Krithika Suresh, Swati G. Patel, Carmen L. Lewis

**Affiliations:** 1 Division of General Internal Medicine, Department of Medicine, University of Colorado School of Medicine, Aurora; 2 Cecil G. Sheps Center for Health Services Research, The University of North Carolina at Chapel Hill; 3 Division of General Internal Medicine and Clinical Epidemiology, Department of Medicine, The University of North Carolina at Chapel Hill; 4 Gillings School of Global Public Health, Department of Health Behavior, The University of North Carolina at Chapel Hill; 5 Division of Data Sciences Safety and Regulatory, Division of Biostatistics, Department of Research & Development Solutions, IQVIA, Durham, North Carolina; 6 Department of Family Medicine, School of Medicine, The University of North Carolina at Chapel Hill; 7 Division of General Internal Medicine, Department of Medicine, Duke University, Durham, North Carolina; 8 Adult and Child Center for Outcomes Research and Delivery Science, University of Colorado Anschutz Medical Campus, Aurora; 9 Department of Biostatistics and Informatics, Colorado School of Public Health, University of Colorado Anschutz Medical Campus, Aurora; 10 Division of Gastroenterology and Hepatology, University of Colorado Anschutz Medical Campus, Aurora; 11 Rocky Mountain Regional Veterans Affairs Medical Center, Aurora, Colorado

## Abstract

**Importance:**

Guidelines recommend individualized decision-making for colorectal cancer (CRC) screening among adults aged 76 to 84 years, a process that includes a consideration of health state and patient preference.

**Objective:**

To determine whether a targeted patient decision aid would align older adults’ screening preference with their potential to benefit from CRC screening.

**Design, Setting, and Participants:**

This is a prespecified secondary analysis from a randomized clinical trial. Participants aged 70 to 84 years who were not up to date with screening and had an appointment within 6 weeks were purposively sampled by health state (poor, intermediate, or good) at 14 community-based primary care practices and block randomized to receive the intervention or control. Patients were recruited from March 1, 2012, to February 28, 2015, and these secondary analyses were performed from January 15 to March 1, 2022.

**Interventions:**

Patient decision aid targeted to age and sex.

**Main Outcomes and Measures:**

The primary outcome of this analysis was patient preference for CRC screening. The a priori hypothesis was that the decision aid (intervention) group would reduce the proportion preferring screening among those in poor and intermediate health compared with the control group.

**Results:**

Among the 424 participants, the mean (SD) age was 76.8 (4.2) years; 248 (58.5%) of participants were women; and 333 (78.5%) were White. The proportion preferring screening in the intervention group was less than in the control group for those in the intermediate health state (34 of 76 [44.7%] vs 40 of 73 [54.8%]; absolute difference, −10.1% [95% CI, −26.0% to 5.9%]) and in the poor health state (24 of 62 [38.7%] vs 33 of 61 [54.1%]; absolute difference, −15.4% [95% CI, −32.8% to 2.0%]). These differences were not statistically significant. The proportion of those in good health who preferred screening was similar between the intervention and control groups (44 of 74 [59.5%] for intervention vs 46 of 75 [61.3%] for control; absolute difference, −1.9% [95% CI, −17.6% to 13.8%]).

**Conclusions and Relevance:**

The findings of this secondary analysis of a clinical trial did not demonstrate statistically significant differences in patient preferences between the health groups. Additional studies that are appropriately powered are needed to determine the effect of the decision aid on the preferences of older patients for CRC screening by health state.

**Trial Registration:**

ClinicalTrials.gov Identifier: NCT01575990

## Key Points

**Question:**

Does a targeted patient decision aid decrease older adults’ preferences for colorectal cancer screening among those unlikely to benefit from it?

**Findings:**

The findings of this secondary analysis of a randomized clinical trial including 424 participants did not demonstrate statistically significant differences in patient preferences between the health state groups. However, compared with the control group, fewer participants in the intervention group preferred screening in the intermediate and poor health states.

**Meaning:**

Additional studies that are appropriately powered are needed to determine the effect of the decision aid on preferences of older patients for colorectal cancer screening by health state.

## Teaser

This secondary analysis of a randomized clinical trial assesses whether a patient decision aid that informs patients about the potential harms would decrease preferences in favor of colorectal cancer screening among older adults unlikely to benefit from it.

## Introduction

Since 2008, the US Preventive Services Task Force has recommended individualized decision-making for colorectal cancer (CRC) screening for adults aged 76 to 84 years,^1^ a process that includes a consideration of health state and patient preferences. Because CRC is typically a slow-growing cancer, the benefits from early detection of polyps accrue 5 to 10 years after undergoing CRC screening.^2,3^ Older adults have a spectrum of health states; therefore, individuals must consider the potential benefit given their particular comorbidities and life expectancy.^3^ For those in poor health with life expectancies less than 5 years, the risks of undergoing CRC screening, which include bleeding, perforation, and death,^4^ likely outweigh the benefits. For older adults in good health, the benefits likely outweigh the risks. Therefore, it is in an older patient’s best interest to be informed about the potential benefits and harms of screening associated with age and comorbidity when making a decision about CRC screening instead of reflexively continuing to undergo screening tests.^3,5,6^

Ideally, individualized decision-making would align screening behavior with the potential to benefit from screening. However, despite these long-standing recommendations, inappropriate CRC screening is a continuing concern among older adults, especially those who are unlikely to benefit from screening.^7,8,9,10^ One driver of such overuse of screening could be some older patients’ preferences to continue to undergo CRC screening despite a low likelihood of potential benefit.^11,12^ However, these preferences in favor of screening may not adequately reflect informed preferences of older adults who, based on their individual health status, are unlikely to benefit from CRC screening.^11,13^ In fact, in-depth interviews with older adults with significant comorbidities indicate that preferences in favor of screening may dissipate as people get older, have more comorbid illnesses, and are informed about the potential harms.^14^

To our knowledge, no randomized clinical trials have examined informed patient preferences for CRC screening among older adults with a spectrum of health states. We performed this analysis to determine whether a patient decision aid that informs patients about the potential harms would decrease preferences in favor of CRC screening among older adults who are unlikely to benefit from it. Specifically, our a priori hypothesis was that the decision aid would reduce the proportion preferring CRC screening among those in poor and intermediate health compared with the control group, but that this reduction would be less pronounced among those in good health.

## Methods

### Study Design and Setting

We performed a prespecified secondary analysis of a randomized clinical trial with 1:1 allocation. The parent study determined that the patient decision aid improved appropriate CRC screening 6 months after the intervention, and the trial was powered on this outcome (Supplement 1).^15,16^ Researchers, study staff performing participant data collection, and patients were blinded to their assignment. We recruited participants from 14 community practices within the Duke Primary Care Research Consortium from March 1, 2012, to February 28, 2015. The study protocol (Supplement 2) was approved by the institutional review boards at Duke University and The University of North Carolina at Chapel Hill. All patients provided written informed consent. We followed the Consolidated Standards of Reporting Trials (CONSORT) reporting guideline.

### Identification of Participants, Eligibility, and Randomization

Participants were eligible if they were aged 70 to 84 years, were not up to date with CRC screening, and had an appointment scheduled within 2 to 4 weeks with a participating practice. We reviewed medical records for eligibility and sent a letter inviting participation and an opt-out card. If patients did not opt out, we contacted them by telephone or in person before their appointment. We confirmed that patients were eligible and obtained consent for them to participate. Other eligibility criteria included English fluency and the ability to use the paper-based intervention. Exclusion criteria included dementia^17^ and any history of CRC or inflammatory bowel disease.

We used purposeful sampling by health state with the goal of recruiting 150 participants in good, intermediate, and poor health states. The health state of potential participants was determined by a combination of age and the adaptation by Deyo et al^18^ of the Charlson Comorbidity Index (CCI). We expected participants in the best health state (aged 70-74 years with a CCI of 0-3 or aged 75-79 years with a CCI of 0) to have a life expectancy of at least 10 years and thus have a net benefit from screening. We expected participants in the intermediate health state (aged 70-74 years with a CCI ≥4, aged 75-79 years with a CCI of 1-3, or aged 80-84 years with a CCI of 0) to have a life expectancy of 5 to 10 years; in this case, the net benefit from screening is uncertain. We expected participants in poor health (aged 75-79 years with a CCI ≥4 or aged 80-84 years with a CCI ≥1) to have a life expectancy of less than 5 years^19^ and thus be unlikely to benefit from screening.^9,10,20,21,22,23,24,25,26^

Participants were randomly assigned through a centralized computer process to either the intervention or control group using block randomization by health state. They received a prelabeled, opaque, sealed packet that was sorted and assembled by research assistants not involved with data collection. Patients’ invitation to the study described a study about preventive services but did not describe the intervention or control materials; thus, patients were blinded to allocation.

### Intervention and Control Materials

As described in prior work,^16,27^ we developed the decision aid intervention “Making a Decision About Colon Cancer Screening” according to the international standards for decision aid development.^28^ The decision aid is a 20-page paper-based tool targeted to patients according to their age and sex.^15,16,27^ The decision aid included (1) a description of stool-based testing, (2) a description of colonoscopy, (3) information explaining that all positive stool study results require a colonoscopy, (4) potential harms of colonoscopy, (5) importance of competing causes of mortality in older patients, and (6) the need to weigh the harms and benefits of colonoscopy for each individual. The decision aid was targeted to age groups (70-74, 75-79, and 80-84 years) and sex because competing causes of mortality varied significantly between these groups, resulting in 6 versions of the decision aid. Participants randomized to the attention control received a driving decision tool developed by the American Automobile Association Foundation for Traffic Safety, entitled “Drivers 65 Plus: Check Your Performance.”^29^

### Conceptual Framework for Intervention

Individualized decision-making frameworks posit that screening preferences will better align with individuals’ potential to benefit when individuals are adequately informed about the harms and benefits and given the opportunity to consider their personal values.^3,5,30^ As with traditional decision aids, we expected an increase in participants’ knowledge and clearer values with decision aid use.^31^ Importantly, we informed them of the uncertainty involved in trying to predict on an individual level who would die and thus not benefit from screening vs who would live long enough to potentially benefit. Finally, we provided a visual bottom line or “gist message”^32^ for each of the 3 health states using an illustration of a scale: the scale demonstrated that the benefits outweighed the harms for the good health state; the scale was balanced for the intermediate health state; and the harms outweighed the benefit in the poor health state. We did not explicitly tell participants their health state within the decision aid.

### Data Collection, Measures, and Outcomes

Patient demographic characteristics and health literacy (REALM [Rapid Estimate of Adult Literacy in Medicine]–Short Form)^33^ were collected during the eligibility and baseline surveys via telephone or in person before the visit. Self-reported race and ethnicity data were collected because members of racial and ethnic minority groups have been underrepresented in previous decision aid studies. Age and comorbidities to determine the aforementioned health states (good, intermediate, or poor) were obtained using the Deyo et al^18^ adaption of the CCI. After interacting with the intervention or control materials but before seeing their clinician, participants completed a questionnaire that asked their screening preference (prefer to get screened, prefer not to get screened, or unsure). The primary outcome was defined as a binary indicator of preference for screening vs no screening or unsure. Secondary decision-making outcomes collected from this questionnaire were patient knowledge and values clarity. Patient knowledge about colon cancer was measured with 5 true-or-false questions and scored as the count of items answered correctly (range, 0-5).^27^ We measured values clarity using the values clarity subscale of the decisional conflict scale.^34^

### Statistical Analysis

Data were analyzed from January 15 to March 1, 2022. Descriptive statistics (means [SDs], frequencies, and percentages) were computed overall and by study group. Baseline participant characteristics were compared between the intervention and control groups using unpaired 2-tailed *t* tests for continuous variables and χ^2^ or Fisher exact tests for categorical variables. The primary analysis compared the proportions of patient preferences for screening between the study groups overall using a linear probability model. In secondary analyses, mean knowledge scores were compared between study groups overall using a Poisson regression model for count data, and mean clarity scores were compared between study groups overall using a linear regression model. To estimate outcome differences between study groups within each health state (ie, to assess for effect modification by health state), models with an interaction term between health state and study group were also evaluated. For each comparison (overall and within each health state), we report the difference between study groups and the 95% CI.^35^ Models accounting for clustering by physician are not presented because they were found to have similar results. All analyses were completed using SAS, version 9.4 (SAS Institute Inc), with the significance level set at α = .05 for 2-sided statistical tests.

## Results

**Figure 1.**
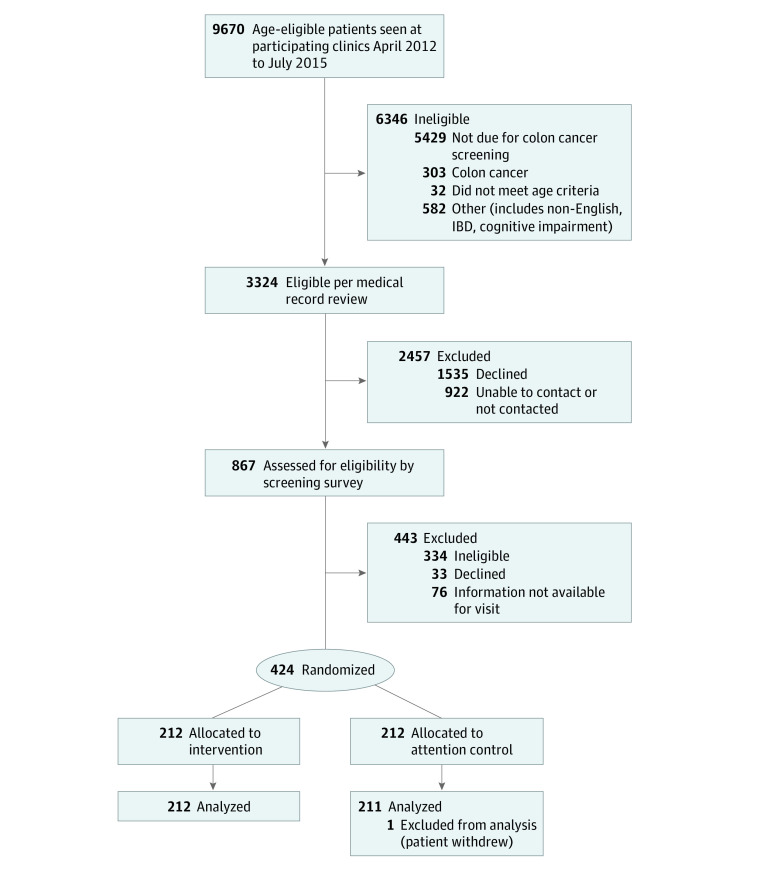
Study Flow Diagram IBD indicates inflammatory bowel disease.

We identified 9670 age-eligible patients with upcoming appointments, among whom 3324 were eligible after medical record review and were sent an invitation to participate (Figure 1). After accounting for exclusions, inability to contact patients, and patients who declined, 424 participants were randomized.

Among the 424 participants, the mean (SD) age was 76.8 (4.2) years; by self-report, 248 participants (58.5%) were women and 176 (41.5%) were men. In terms of race and ethnicity, 1 participant (0.2%) was American Indian or Alaska Native; 7 (1.7%) were Asian; 74 (17.5%) were Black; and 333 (78.5%) were White. Participants were highly literate: 357 of 402 with available data (88.8%) scored a 7 of 7 on the REALM–Short Form^33^ (Table 1).

**Table 1.** Participant Characteristics

Characteristic	Participant group, No. (%)^a^		
	All (n = 424)	Intervention (n = 212)	Control (n = 212)
Age, mean (SD)	76.8 (4.2)	76.6 (4.1)	77.0 (4.3)
Sex			
Women	248 (58.5)	130 (61.3)	118 (55.7)
Men	176 (41.5)	82 (38.7)	94 (44.3)
Race and ethnicity			
American Indian or Alaska Native	1 (0.2)	1 (0.5)	0
Asian	7 (1.7)	1 (0.5)	6 (2.9)
Black or African American	74 (17.5)	35 (16.5)	39 (18.6)
Native Hawaiian or other Pacific Islander	0	0	0
White	333 (78.9)	173 (81.6)	160 (76.2)
Multiple^b^	3 (0.7)	0	3 (1.4)
Other^c^	4 (0.9)	2 (0.9)	2 (1.0)
Hispanic or Latino ethnicity	7 (1.7)	3 (1.4)	4 (1.9)
Educational status			
Some high school or less	30 (7.1)	15 (7.1)	15 (7.1)
High school graduate	95 (22.4)	58 (27.4)	37 (17.5)
Some college or associate’s degree	97 (22.9)	44 (20.8)	53 (25.0)
Bachelor’s degree	90 (21.2)	40 (18.9)	50 (23.6)
Advanced degree	112 (26.4)	55 (25.9)	57 (26.9)
Marital status			
Married or cohabiting	241 (56.8)	117 (55.2)	124 (58.5)
Widowed	100 (23.6)	53 (25.0)	47 (22.2)
Divorced	63 (14.9)	32 (15.1)	31 (14.6)
Never married or other	20 (4.7)	10 (4.7)	10 (4.7)
Primary health insurance			
Medicare	378 (90.2)	189 (89.6)	189 (90.9)
Medicaid	6 (1.4)	2 (9.4)	4 (1.9)
Private	14 (3.3)	9 (4.3)	5 (2.4)
Employee or union	13 (3.1)	6 (2.8)	7 (3.4)
Other	8 (1.9)	5 (2.4)	3 (1.4)
Work status			
Retired	354 (83.7)	174 (82.1)	180 (85.3)
Currently employed	58 (13.7)	32 (15.1)	26 (12.5)
Unemployed	11 (2.6)	6 (2.8)	5 (2.4)
Ever been screened for CRC	377 (88.9)	187 (88.2)	190 (89.6)
No. of health literacy items correct^d^			
7	357 (88.8)	178 (90.8)	179 (86.9)
0-6	45 (11.2)	18 (9.2)	27 (13.1)
Self-reported health			
Excellent	44 (10.4)	25 (11.8)	19 (9.0)
Very good	136 (32.1)	62 (29.2)	74 (34.9)
Good	168 (39.6)	85 (40.1)	83 (39.2)
Fair	67 (15.8)	36 (17.0)	31 (14.6)
Poor	9 (2.1)	4 (1.9)	5 (2.4)
Saw usual physician at study visit	407 (97.6)	207 (98.6)	200 (96.6)
Primary reason for visit			
Routine check-up	218 (51.5)	111 (52.4)	107 (50.7)
Follow-up	186 (44.0)	88 (41.5)	98 (46.4)
New problem	11 (2.6)	7 (3.3)	4 (1.9)
Another reason	8 (1.9)	6 (2.8)	2 (0.9)

Abbreviation: CRC, colorectal cancer.

^a^ Owing to missing data, numbers of participants for some categories may not sum to column totals.

^b^ Includes 1 participant who reported American Indian or Alaska Native and Black; 1 who reported Asian, Native Hawaiian or other Pacific Islander, and White; and 1 who reported Black, American Indian or Alaska Native, and White.

^c^ Includes those who self-reported other race or ethnicity.

^d^ Twenty-two participants (5.2%) of the total sample had missing responses on the health literacy test; maximum score was 7.

### Decision-making Outcomes

**Figure 2.**
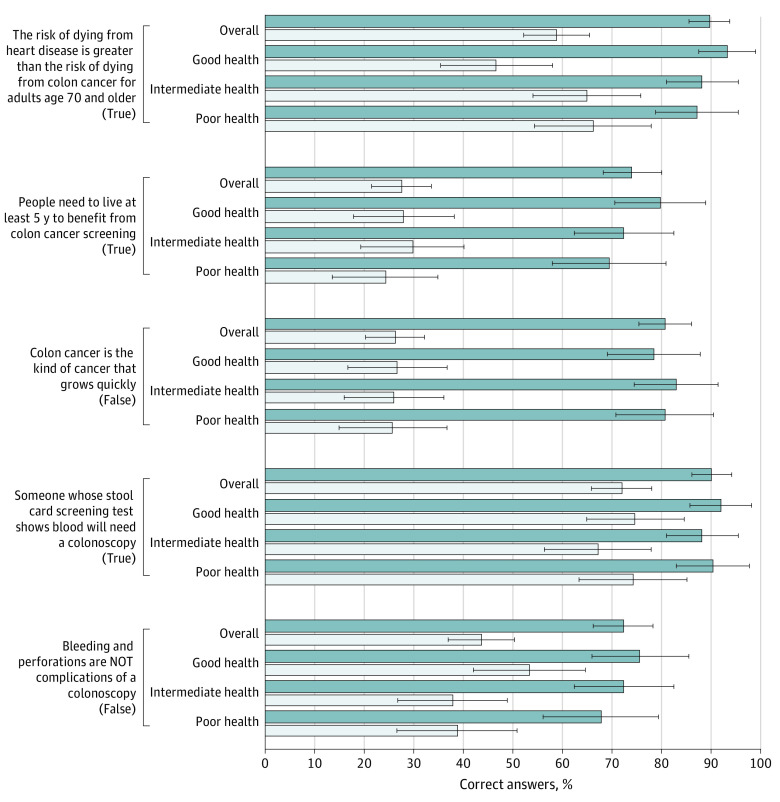
Responses to Knowledge Questions by Intervention Group and Health State Data are expressed as mean (SD); error bars indicate the SD.

Participants in the intervention group scored statistically significantly higher on the CRC knowledge questions than participants in the control group (mean [SD], 4.1 [1.1] vs 2.3 [1.2]) (Table 2 and Figure 2). The differences in knowledge scores between the intervention and control groups were similar across health states (no significant interaction effect). The intervention group had a lower mean score on the values clarity subscale (mean [SD], 2.33 [15.4] vs 26.9 [17.9]), representing greater clarity. This difference was similar within each of the health states (no significant interaction effect) (Table 2).

**Table 2.** Knowledge, Values Clarity, and Patient Screening Preference by Intervention Group and Health State

Measure	Intervention group		Control group		Difference, estimate (95% CI)	*P* value
	No. of participants	Estimate	No. of participants	Estimate		
**CRC knowledge score, mean (SD)**^a^						
Overall	212	4.1 (1.1)	211	2.3 (1.2)	1.8 (1.6 to 2.0)	<.001^b^
By health state						.92^c^
Good	74	4.2 (1.1)	75	2.3 (1.2)	1.8 (1.5 to 2.2)	<.001^d^
Intermediate	76	4.0 (1.0)	74	2.2 (1.3)	1.8 (1.5 to 2.2)	<.001^d^
Poor	62	4.0 (1.2)	62	2.3 (1.2)	1.7 (1.4 to 2.1)	<.001^d^
**Values clarity subscale score, mean (SD)**^e^						
Overall	212	23.3 (15.4)	210	26.9 (17.9)	−3.6 (−6.8 to −0.4)	.03^b^
By health state						.90^c^
Good	74	22.1 (15.4)	75	26.1 (17.7)	−4.0 (−9.4 to 1.4)	.14^d^
Intermediate	76	23.6 (15.5)	73	27.7 (19.1)	−4.2 (−9.6 to 1.2)	.13^d^
Poor	62	24.3 (15.5)	62	26.7 (16.8)	−2.4 (−8.3 to 3.5)	.42^d^
**Preference for screening, No. (%)**						
Overall	212	102 (48.1)	209	119 (56.9)	−8.8 (−18.3 to 0.7)	.07^b^
By health state						.52^c^
Good	74	44 (59.5)	75	46 (61.3)	−1.9 (−17.6 to 13.8)	.82^d^
Intermediate	76	34 (44.7)	73	40 (54.8)	−10.1 (−26.0 to 5.9)	.22^d^
Poor	62	24 (38.7)	61	33 (54.1)	−15.4 (−32.8 to 2.0)	.08^d^

Abbreviation: CRC, colorectal cancer.

^a^ Scores range from 0 to 5, with higher scores indicating more knowledge.

^b^ *P* value from main effect model (no interaction).

^c^ *P* value for interaction effect from model with interaction between health state and study group.

^d^ *P* value from model with interaction between health state and study group.

^e^ Scores range from 0 to 100, with higher scores indicating clearer values.

### Screening Preferences

**Figure 3.**
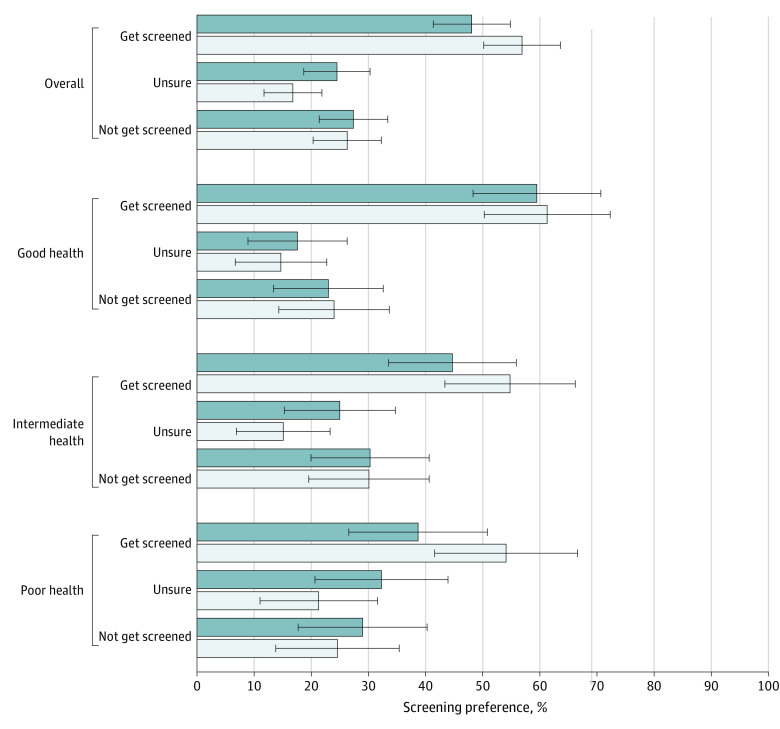
Participant Preferences for Screening by Intervention Group and by Health State Data are expressed as mean (SD); error bars indicate the SD.

Overall, the proportion of participants who preferred screening in the intervention group was less than in the control group, but these differences were not statistically significant (102 of 212 [48.1%] vs 119 of 209 [56.9%]; absolute difference, −8.8% [95% CI, −18.3% to 0.68%]; *P* = .07) (Table 2 and Figure 3). The proportion who preferred screening in the good health state was similar between the intervention and control groups (44 of 74 [59.5%] for intervention vs 46 of 75 [61.3%] for control; absolute difference, −1.9% [95% CI, −17.6% to 13.8%]; *P* = .82). The proportion preferring screening among those in the intervention group was less than among those in the control group in the intermediate health state (34 of 76 [44.7%] vs 40 of 73 [54.8%]; absolute difference, −10.1% [95% CI, −26.0% to 5.9%]; *P* = .22) and in the poor health state (24 of 62 [38.7%] vs 33 of 61 [54.1%]; absolute difference, −15.4% [95% CI, −32.8% to, 2.0%]; *P* = .08). There was no statistically significant interaction effect between health state and study group. A higher proportion of participants in the intervention group reported being uncertain about screening preferences (Figure 3).

## Discussion

As with other decision aid trials,^31^ we found that the targeted decision aid for CRC screening in older adults improved knowledge and helped clarify their values before an appointment with their clinician, suggesting that participants understood the message in the decision aid that screening may not be beneficial for all older adults and were better prepared to make a decision. We did not demonstrate statistically significant differences in patient preferences between study groups by health state in this secondary analysis. However, the decrease in the proportion of participants in intermediate and poor health states who preferred screening after engaging with the decision aid compared with those who received the control materials was consistent with our a priori hypothesis and may be clinically meaningful. The decision aid group also had an increase in the proportion of participants who were undecided about their screening preference, also suggesting the information may have had an effect on patient preference.

To our knowledge, few decision aids have been developed for older adults making decisions about cancer screening. Schonberg et al^36^ demonstrated a decrease in mammography screening using a decision aid for women 75 years and older. Our approach went beyond this study in that we stratified our sample into good, intermediate, and poor health states so that we could examine whether the decision aid aligned screening preferences with the potential to benefit from screening. Using decision aids for this purpose is novel because it promotes an individualized decision, rather than shared decision-making. The distinction is subtle but important. Traditionally, the shared decision-making framework promotes “preference-sensitive decision-making,” meaning that physicians are at equipoise and preferences of the patient drive the decision. In contrast, using an individualized decision-making approach, physicians consider the risks and benefits of screening given a person’s health state and life expectancy.^37,38,39^ They may not be at equipoise, and in fact, may be concerned about net harms for patients undergoing colonoscopy without a potential benefit. Consequently, we designed our decision aid so that older adults consider their personal preferences in light of the risks and benefits given their particular health state. Consistent with libertarian paternalism, we did not forbid any options, but the decision aid encouraged older adults to consider their options and provided them with information that might nudge them toward an option that is in their best interest.^40^

From the patient perspective, use of a decision aid may be able to modify older adult preferences to align with the potential to benefit from screening. Studies surveying older adults who are primarily in good health about their screening preferences indicate that many would like to continue screening.^12,41,42,43^ We found a potentially clinically meaningful reduction in the proportion of participants in the intervention group who preferred to undergo screening in the intermediate and poor health states compared with the control group, although these differences were not statistically significant. Furthermore, a higher proportion in the intervention group reported being uncertain about their screening preference. These results are not definitive but may suggest that patients understood the messages in the decision aid that CRC screening may not benefit patients with limited life expectancy, and that estimating life expectancy for individuals involves clinical uncertainty. Our study is important because it demonstrates that, among a sample of older adults with a broad range of health states, when informed and faced with the screening decision, older adults’ preferences may differ based on health state and life expectancy. Our findings may also support the use of an individualized decision-making framework in older adults as recommended by the US Preventive Services Task Force^3,44^ by demonstrating that patient preferences in favor of screening may change once patients are adequately informed. Additionally, our results help to offset concerns that older adults may perceive recommendations against screening as rationing care. Instead, older adults may prefer not to undergo screening because it is in their best interest to avoid it.

Modifying the choice architecture away from the default option of screening for younger, healthy adults, as we did in our CRC decision aid, may be beneficial for a broader array of decisions faced by older adults with limited life expectancy.^45^ As recommended by the International Patient Decision Aid Standards^46^ at the time we were developing the decision aid, we designed our decision aid to have the capability to engage patients in a deliberative mode of decision-making—ie, so-called central processing or system 2 thinking.^47^ To do so, we provided icon arrays for the risks and benefits of undergoing screening in addition to the risk of dying of colon cancer compared with other common health conditions. Because the risk of dying of these other common conditions varied significantly by age and sex, we provided decision aids targeted by age range and sex. The targeting was designed to provide patients with accurate information to achieve informed preferences. Although targeting by age may enhance the relevance of information to older adults,^48^ we did not assess its effect in this study.

Evidence suggests that combining deliberative and intuitive decision-making processes may improve the effectiveness of decision aids.^49^ Furthermore, older patients may differ with regard to decision-making processing,^50^ and they may rely more frequently on gist-based decision-making.^51^ Therefore, we provided a gist summary^32,52^ that used a scale to balance the risks and benefits for each health state.

### Limitations

This study was limited by the lack of statistical power to assess screening preference differences overall and by health state. Our results demonstrated a decrease in the proportion preferring screening with a worsening health state, which we consider to be a clinically meaningful result.^53^ However, additional studies that are appropriately powered will be necessary to confirm the effect of the decision aid by health state with statistical significance. In this efficacy study, the participants were primarily White and well-educated, limiting the generalizability of our results. Furthermore, most of the participants had been screened previously, and we are uncertain as to how preferences for screening might change among those who had not undergone screening. The study was performed in 2013 before the widespread use of fecal immunochemical testing. Although the decision aid presented information about stool-based testing and demonstrated that a positive test would result in the need for colonoscopy, we are unsure how patient preferences would change with regard to newer screening tests.

## Conclusions

The findings of this secondary analysis of a randomized clinical trial did not demonstrate statistically significant differences in patient preferences between health state groups. The proportion of participants in intermediate and poor health states who preferred screening decreased after engaging with the decision aid compared with those who received the control materials, but these differences were not statistically significant. Additional studies that are appropriately powered are needed to determine the effect of the decision aid on preferences of older patients for CRC screening by health state.

## Notes

Supplement 1.
Research Strategy

Supplement 2.
Trial Protocol

Supplement 3.
Data Sharing Statement
