# Supplementary material for: Effect of a Patient Decision Aid on Preferences for Colorectal Cancer Screening Among Older Adults: A Secondary Analysis of a Randomized Clinical Trial
Source: JAMA Netw Open. 2022 Dec 5;5(12):e2244982. doi: 10.1001/jamanetworkopen.2022.44982 (PMC9855297; doi:10.1001/jamanetworkopen.2022.44982)
Supplement: Supplement 1. — Research Strategy [file jamanetwopen-e2244982-s001.pdf]

RESEARCH STRATEGY

Overall Strategy

The overall goal of our Reducing Potential Harms of Clinical Preventive Services (ReCPS) Center is to lead a major effort to reduce the potential harms of USPSTF-rated C, D, and I clinical preventive services by increasing appropriate decisions. Our strategy relates directly to our Overall Specific Aims. The first 2 of these Aims involve increasing attention to and improving the quality of research on this issue. The third Aim involves increasing the visibility of the issue of overuse among physicians and policy makers through (1) a model educational program to current and future physicians, and (2) a strong dissemination program directed to physicians and policy makers. Both education and dissemination programs will be based on what is already known about potential harms as well as new knowledge developed by our and other research projects. Our strategy for achieving these Aims involves developing a strong Core Office as well as a strong research program.

Core Office

Administrative and Organizational Structure

The ReCPS Core Office will coordinate activities that cross research projects, create an innovation program to develop and disseminate new research methods and concepts, coordinate activities of the ReCPS Roundtable and Review Committee, house shared resources for the research projects, and lead the Center's activities in training, education, and dissemination. The Core Office will be physically located in the UNC-Cecil G. Sheps Center for Health Services Research. The organizational chart is shown in Figure 1.

Figure 1: UNC Research Center for Excellence in Clinical Preventive Services (ReCPS)

Organizational Chart

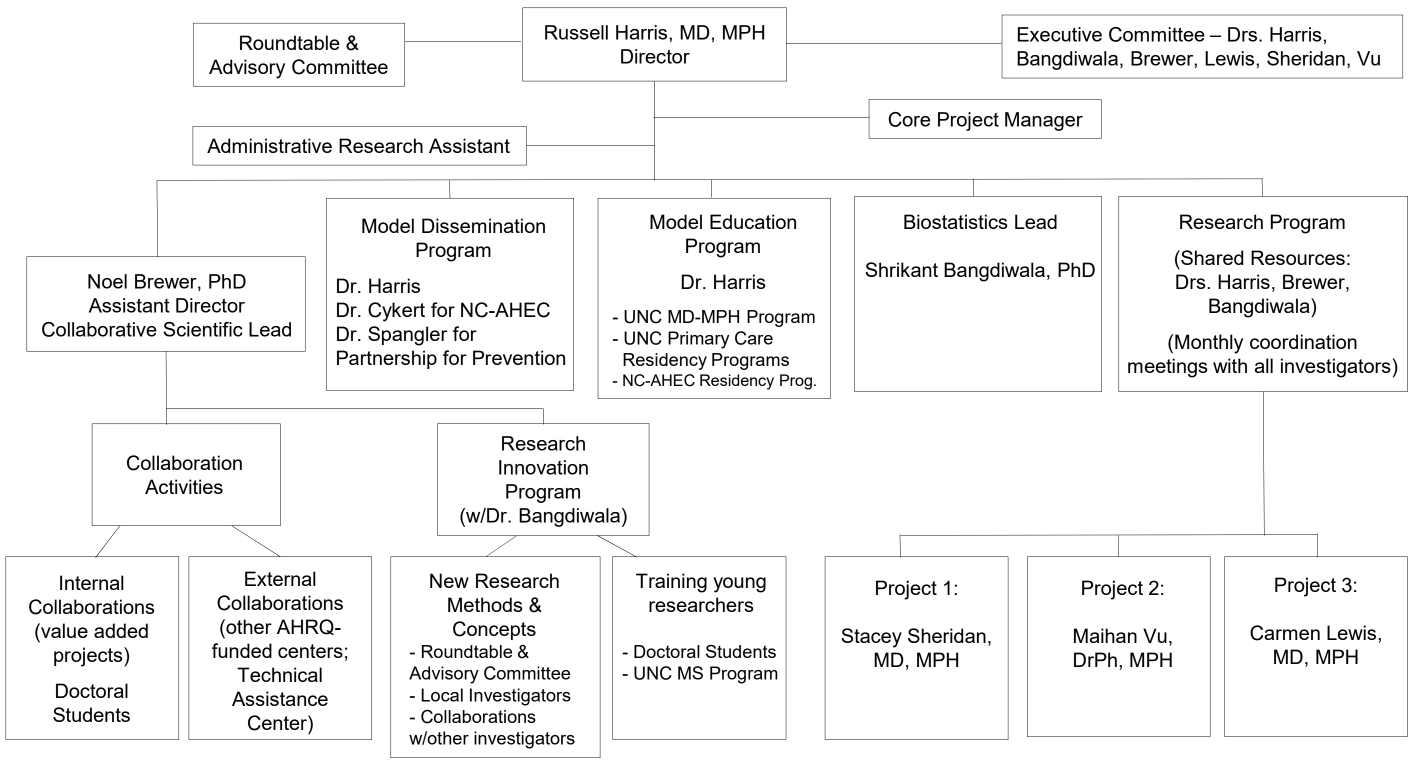

Center Personnel

The Center Director will be Russell Harris, MD, MPH. Dr. Harris will oversee the entire center, lead the education program, and coordinate the dissemination program. The Scientific Collaborative Lead and Assistant Center Director will be Noel T. Brewer, PhD, MS. Dr. Brewer will lead collaboration of UNC-ReCPS with other grantees of this RFA and with the Technical Assistance Center. He will also lead the research innovation program in developing and disseminating new research methods and concepts as well as the Center's work in training young researchers in research methods.

Drs. Harris and Brewer have extensive experience in the problem of overuse of potentially harmful clinical preventive services in primary care practice. Dr. Harris is Professor of Medicine and Adjunct Professor of Epidemiology at UNC; he leads the UNC MD-MPH Program. He has practiced primary care both in

community and academic settings for the past 40 years and has been Project Director or Co-Principal Investigator of 4 NCI or CDC – funded projects in community based primary care practices since becoming a faculty member at UNC some 25 years ago. Dr. Harris led the UNC section of the RTI-UNC Evidence-based Practice Center from 1997 to 2002 in conducting systematic reviews for the US Preventive Services Task Force (USPSTF), making regular presentations to the Task Force. From 2003 to 2008, Dr. Harris was a member of the USPSTF, leading recommendation discussions for multiple topics. From 1997 to 2008, he was an active member of the Methods Work Group of the USPSTF, and participated in several methods publications. His methodological interests include overdiagnosis and the effects of false positive screening tests.

Dr. Brewer is an experienced theorist and decision psychologist. He is an expert on medical decision-making with an emphasis on risk communication about medical tests and vaccinations. He is Associate Professor of Health Behavior and Health Education in the UNC Gillings School of Global Public Health. Dr. Brewer is a member of the US Food and Drug Administration's Risk Communication Advisory Board. He has published several widely-cited systematic reviews and meta-analyses on preventive services, including one in 2007 on the effects of false positive mammograms on psychological health and on subsequent screening behavior.

Also in the Core Office will be Shrikant Bangdiwala, PhD, the Biostatistics Lead. Dr. Bangdiwala also has extensive experience with community-based observational studies and intervention trials. He will work with Dr. Brewer in the research innovation program, including collaborating with the Technical Assistance Center, and participating in training new researchers in methods and concepts, adding his expertise in biostatistics and research design methods. Drs. Harris, Brewer, and Bangdiwala will serve as shared resources for all research projects housed in the Center.

All investigators for studies housed in the Center will be included in the Core Office. To promote coordination and development of new methods and ideas, the Collaborative Scientific Lead will organize monthly Coordination Meetings, including presentations from inside and outside of UNC. These sessions are meant to serve several functions, including collaboration and integration among the projects, research training, and education, as well as problem-solving as needed.

Planning, Assessment of Progress, and Evaluation

The Core Office will organize quarterly Executive Meetings led by Dr. Harris and including Drs. Brewer, Bangdiwala, Lewis, Sheridan, and Vu. The meeting agenda will include (a) assessment of the progress of the Core Office programs (research innovation and training, collaboration, education, dissemination); (b) Center evaluation, including creating measurable indicators of success for each program, with ongoing measurement and monitoring of the indicators; and (c) planning for improvement and for future development. The Core Office will produce an "Assessment and Evaluation" report once each year for the Roundtable and Advisory Committee. The Executive Committee will review and implement suggestions from the Roundtable and Advisory Committee, as practicable.

Collaborative Activity and Research Innovation Program

Dr. Brewer will serve as the Collaborative Scientific Lead, supervising external and internal activities related to research innovation. In coordinating among external partners, he will communicate and collaborate in a timely and interactive way with the other 2 AHRQ-funded centers, the Technical Assistance Center, and AHRQ on issues related to patient safety. Activities will also include preparing presentations for cross-center meetings; developing research agendas or other recommendations for future assessment, intervention, and evaluation; and drafting publications around patient safety innovations.

Internal activities will, together with the Core Office doctoral student, focus on creating one or more small value-added research studies that will be embedded in the 3 research projects. This value-added study will reflect, in part, ideas from the ReCPS Roundtable and Advisory Committee. Findings from this study will be submitted for publication in a peer-reviewed journal. For example, this study could focus on how patients' and physicians' understand false positive screening test results, or the concept of overdiagnosis. These small "value-added" projects could also pilot test new research methods. Public health doctoral students will work with Dr. Brewer on these projects, training in research methods and skills.

Integrated with the collaborative activities is the Research Innovation Program. The purpose of this program is to assess the current state of theories and methods used in research concerning potential harms and overuse, and to make suggestions about improving these theories and methods. One of the systematic reviews that will be conducted by the Core Office will catalogue the theories and methods used in studies of potential harms and overuse. Dr. Brewer will reach out to other research centers and members of the ReCPS

Roundtable and Advisory Committee to critically appraise these theories and methods and develop innovations to improve our research in this area. Specific areas of focus may include establishing a basis in behavioral theory for encouraging people to not engage in a health behavior, as well as developing approaches for understanding the harms of screening overuse.

Model Dissemination Program

Dr. Harris will coordinate the dissemination program, working closely with the North Carolina Area Health Education Centers (NC AHEC) and its Regional Extension Center (REC), and with the national nonprofit group Partnership for Prevention. Samuel Cykert, MD, will represent NC AHEC and Jason Spangler, MD, MPH, will represent Partnership for Prevention within the Core Office. Together we will develop a strong dissemination effort, working to increase the attention paid in both clinical practice and in policy making to the potential harms of clinical preventive services. This double-pronged approach to dissemination provides ReCPS with both a local "laboratory" for dissemination (i.e., the NC-AHEC Program) and a means for "scaling up" effective dissemination strategies by working with the Partnership for Prevention.

Samuel Cykert, MD, Associate Director for Medical Education of the NC AHEC Program and Clinical Director of the REC, will be a member of the Core Office. The REC was established with a federal grant to support primary care providers in adopting electronic health records and using health information technology effectively to improve the quality of health care. The NC AHEC has a 37 year history of establishing community-based training programs for health professionals. Over the past 5 years, NC AHEC has developed the REC, a nationally-recognized model for improving care by providing hands-on, ground level quality improvement consulting and support in primary care practices throughout the state. To date, 750 primary care practices with 2800 providers have signed up for REC services; another 250 practices and 1200 providers are expected to join by the end of 2011. The NC AHEC and its REC function will serve the dissemination role of ReCPS with a multimodal approach. First, AHEC primary care residency programs (3 internal medicine, 7 family medicine, totaling over 200 residents and 80 full-time faculty) will serve as pilot sites and learning laboratories for the educational content regarding reducing the harms of clinical preventive services. Second, the health information technology environment and the NC AHEC quality improvement mechanism of the REC will be used to engage participating practices to employ the concepts and strategies developed by the ReCPS and translate them into actual practice. Measures of potentially harmful preventive care could be adopted as part of the electronic health record infrastructure, and the rapid cycle quality improvement arm of the REC could then be used to develop ways for providers to reduce the potential harms of preventive services.

Partnership for Prevention (Partnership) will also be a member of our Core Office, as well as hold membership on the ReCPS Roundtable and Advisory Committee. Partnership is a national nonprofit group, founded in 1991 to make disease prevention and health promotion a national priority. Over its 20 year history, Partnership has earned wide praise for bringing together diverse stakeholders, facilitating dialogue on critical issues to find mutually agreeable solutions. Jason Spangler, MD, MPH, Chief Medical Officer at Partnership for Prevention, will represent Partnership of the Core Office. Dr. Spangler carries out Partnership's strategic plan, including its policy agenda; assists in the execution of Partnership projects and initiatives; assures the organization's adherence to scientific principles; and represents Partnership at various conferences, expert panels, and advisory groups. With the advice of the Roundtable and Advisory Committee, Partnership and ReCPS together will develop a strategy of dissemination to policy-makers, including professional associations, business groups, and developers of quality standards. Together, we will create presentations and engage in discussions to find ways to increase the visibility of the issue of overuse of potentially harmful clinical preventive services.

ReCPS Roundtable and Advisory Committee

The Core Office will also organize a Roundtable and Advisory (R and A) Committee that will meet in person once each year and by conference call at least one additional time each year. The members of the R and A Committee are nationally known experts in decision making, psychological theory, research methodology, communication, and prevention. The following people have agreed to be members: Neil Weinstein, PhD; Steven Woolf, MD, MPH; Steven Woloshin, MD; Lisa Schwartz, MD; Jason Spangler, MD, MPH; George Sawaya, MD; Valerie Reyna, PhD; Timothy Carey, MD, MPH; Michael Pignone, MD, MPH, and David Ransohoff, MD. The R and A Committee will advise on the design and examine the results of 3 systematic reviews of the literature on potential harms and overuse conducted during the first 18 months of ReCPS by Dr. Harris and his MPH students. The 3 systematic reviews will: (1) update previous USPSTF reviews on the magnitude and certainty of specific harms for C, D, and I services for the 4 ReCPS exemplar preventive services and up to 4 additional C, D, and I services with public health importance, summarizing

research methods across topics; (2) determine how what is known about the potential harms of these services is being used by primary care practices in decision-making; and (3) examine the effectiveness of various communication (or other) strategies in stimulating discussion of potential harms between clinicians and patients. The R and A Committee will discuss these reports, recommend new research that is needed, and recommend new methods that should be developed. The R and A Committee will also consider the issue of measurement and monitoring of overuse of potentially harmful clinical preventive services. Finally, the R and A Committee will review an annual Assessment and Evaluation Report from the ReCPS Center, making suggestions for improvement and expansion.

Model Education Program

Dr. Harris will lead the model education program. Dr. Harris leads the MD-MPH program at the UNC Schools of Medicine and Public Health, with 45-50 new students each year. About 35 of these students are medical students (UNC, Duke, and other schools) and the other 10-15 are fellows and junior faculty. These students take a year out of their usual training to pursue an MPH. The required courses for the MPH degree include "Strategies of Prevention for Physicians" (led by Dr. Harris) and "Critical Appraisal of the Health Literature" (led by Drs. Harris and Sheridan), as well as additional courses in health policy, clinical epidemiology, and biostatistics. Additionally, a popular elective course on communication for health related decision making (led by Dr. Sheridan) teaches strategies for designing and testing decision making approaches, and the skills needed to implement them.

The ReCPS Center will provide an important focus for education for these students, including attending seminars organized by the Center, and undertaking Master's Papers and practica within the Center. One major focus will be working with Dr. Harris to conduct the 3 systematic reviews outlined above (Roundtable and Advisory Committee section). These reviews will be conducted in a staggered fashion over the first 18 months of the ReCPS project. As noted, they will be presented to the R and A Committee and will be submitted for publication.

In addition, faculty in the MD-MPH Program will work with ReCPS personnel to develop curricular materials designed for use in medical schools and residency programs. We will pilot these materials in the MD-MPH program and in the primary care residency programs at UNC and in the NC-AHEC. After these pilots, we will post the curricular materials for general use by others, and promote the materials at national meetings. If funding is available, we will also organize webinars of this material.

We will evaluate our educational program through monitoring use of curricular materials and conducting surveys of students. These reports will be submitted regularly to the R and A Committee for its suggestions and feedback.

Research Program

We propose 3 integrated research projects to better understand how primary care patients and physicians think about the potential harms of clinical preventive services; how they use their understanding to make decisions about use of these services; and the effects of a decision support system on decision making. These projects are summarized in Table 1.

Table 1: ReCPS Reducing Potential Harms of Clinical Preventive Services

Project Leader	Project Title	Research Design and Focus	Integration with Other Projects
Project 1: Stacey Sheridan, MD, MPH	Understanding and Discouraging Overuse of Potentially Harmful Screening Tests	- Qualitative interviews with patients; whether and how patients conceptualize harms of preventive services<br>- Randomized controlled trial of 4 different presentations of harms; understand effects of various presentations on intent to be screened	- Triangulates patient interview results with results of physician interviews in Project 2<br>- Examines patient factors influencing decisions about CRC screening at ages 75-85, providing perspective on Project 3
Project 2: Maihan Vu, DrPH, MPH	Understanding Physicians' and Patients' Views of Harms and Clinical Preventive Services	- Quantitative survey of all physicians; general understanding of beliefs, attitudes toward harms of exemplar services<br>- Qualitative interviews with selected physicians; deeper understanding of beliefs, attitudes toward harms of exemplar services<br>- Case studies of selected, high risk patients and physicians from Project 3; deeper understanding of decision making	- Triangulates physician interviews with patient interviews in Project 1<br>- Provides deeper understanding of results of decision support intervention in Project 3, as physician attitudes and beliefs may have impact on how patients respond to intervention
Project 3: Carmen Lewis, MD, MPH	Improving Appropriate Colorectal Cancer Screening in Elderly Patients	- Randomized controlled trial of patient decision support intervention; understand effects of decision support on screening decisions and screening completion	- Project 2 will conduct case studies on patients at high risk of harms and their physicians to understand decision making in more depth

These projects are integrated in several ways. First, they are focused on different aspects of the same general problem: the potential harms associated with overuse of USPSTF C, D, and I – rated services. Thus, together they provide different pieces to the puzzle of how to increase appropriate decision-making about these types of services. Secondly, the projects interact in various ways (Table 1), each providing information to allow a deeper interpretation of the results of the other projects. Thirdly, the projects study the same 4 exemplar clinical preventive services, each rated C, D, or I by the USPSTF, chosen because of evidence of overuse, the variation in populations affected, and variation in the magnitude and certainty of the harms involved. Table 2 briefly reviews these services. Fourthly, the 3 projects study medical practices in the same practice-based research network. Thus, our projects will be able to develop an overall picture of decision-making about these C, D, and I services in these practices that will be useful in future studies of other practices and other services. We will briefly describe the practice-based research network that ReCPS will be working with, and then describe each of the 3 projects in detail.

Table 2: ReCPS Exemplar Clinical Preventive Services

Clinical Preventive Service	USPSTF Recommendation or Statement	Magnitude of Potential Harms	Certainty of Evidence of Harms
Osteoporosis Screening for Women ages 50-64 without fracture risk factors	Only recommends screening for women ages 65 and older (no specific grade given for younger women except those with risk factors; previously this group was C)	- Small due to atypical fractures or GERD from use of bisphosphonates;<br>- Potentially larger if use before 65 makes use after 65 less effective;<br>- Labeling and anxiety are potential harms, uncertain magnitude	- High certainty about small risk of atypical fractures or GERD from use of bisphosphonates;<br>- Low certainty about reduced effectiveness after age 65 due to use at earlier age and about labeling and anxiety
EKG/ETT screening for men and women ages 50 - 85	D for ten-year risk < 10%;<br>I for ten-year risk > 15%	- Moderate from work up due to false positives<br>- Labeling, anxiety, and overdiagnosis are potential harms, uncertain magnitude	- Moderate certainty for false positives<br>- Low certainty for labeling, anxiety, overdiagnosis
PSA screening for men ages 50 - 85	I for men ages 50 to 75;<br>D for men older than 75	- Moderate to high due to work up from false positives, treatment of over-diagnosed cases, complications of treatment	- High certainty
Colon cancer screening for men and women ages 70-85 years	C for ages 76-85;<br>D for ages older than 85	- Moderate to high due to increasing complications from colonoscopy in older people and to increasing overdiagnosis with aging	- Moderate certainty

Our ReCPS research program partners with the Duke Primary Care Research Consortium (PCRC). Eighteen practices in this network provide primary care to adults over age 50 years and would be eligible for participation in our studies. These practices include over 100 family physician or internal medicine primary care physicians. There is low physician turnover within these practices. A recent sample of 14 of these practices had an enrollment of 52,793 patients ages 55 or older. Patient recruitment is also excellent within these practices (88%-91%), with low drop-out rates. Previous studies have demonstrated a mix of socioeconomic groups, including 23% on Medicaid and about 27% are African-American.

The PCRC is organizationally within the Duke University Health System and surrounding communities and consists of a central office that handles contractual and organizational issues for research studies. The PCRC includes dedicated clinical research nurses trained by a central office. Rowena Dolor, MD, MHS, has been the Director of the PCRC since its inception in 1997 and is a co-investigator with the ReCPS program. She has collaborated with UNC on 2 previous AHRQ PBRN Master task orders. She is joined in the central office by an Associate Director and a Project Leader, an RN, BSN who handles day-to-day clinical research administrative duties. The central office is responsible for maintaining the interface with the research team for administrative issues and project timelines. Study recruitment is handled primarily by the central office, with oversight and assistance by the research team, aided by a common electronic health record system across sites. Participation in research projects in this network is outstanding, with no practice ever withdrawing from a study, and physician survey response rates usually above 80%. The PCRC Study Coordinator Group consists of 3 clinical research coordinators and 3 clinical trial assistants. Each of these coordinators covers multiple clinical sites. They are well known to and trusted within each practice.

Table 3: Overall ReCPS Timeline

Task	Year 1				Year 2				Year 3			
**Core**	**Q1**	**Q2**	**Q3**	**Q4**	**Q 1**	**Q2**	**Q3**	**Q4**	**Q1**	**Q2**	**Q3**	**Q4**
Roundtable & Adviosory Committee Meetings												
Integrated Systematic Reviews w/ MPH Program												
Collaborative Work with Centers and Others												
Research & Theory Innovation Development												
"Value-Added" Projects												
Dissemination Plan Development												
Dissemination Plan Implementation												
Curriculum Development												
Curriculum Implementation												
Core Office Evaluation												
Presentations at National Meetings												
Annual Report to Roundtable, Advisors, Funder												
Apply for Continuation/Future Funding												
**Project 1**	**Q1**	**Q2**	**Q3**	**Q4**	**Q 1**	**Q2**	**Q3**	**Q4**	**Q1**	**Q2**	**Q3**	**Q4**
Project Director and RA Hiring												
Practice Recruitment												
Individual interview recruitment and Interviews												
Individual interview analysis												
Survey Development												
Patient recruitment and survey administration												
Data Analysis												
Papers and Presentations												
**Project 2**	**Q1**	**Q2**	**Q3**	**Q4**	**Q 1**	**Q2**	**Q3**	**Q4**	**Q1**	**Q2**	**Q3**	**Q4**
Hire & Train Research Assistant												
Development of Surveys and Interview Guides												
Development of Qualitative Data Management System												
Pilot Test Interview Guides												
Modification of Interview Guides												
Data Collection – Survey and Interviews												
Data Analysis												
Data Integration (with Project 2)												
Data Collection – Survey												
Development of Case Studies (with Project 3)												
Data Analysis, Integration, Report Writing												
Papers and Presentations												
**Project 3**	**Q1**	**Q2**	**Q3**	**Q4**	**Q 1**	**Q2**	**Q3**	**Q4**	**Q1**	**Q2**	**Q3**	**Q4**
Start up												
Practice and Physician Recruitment												
Training Research Staff												
Patient Recruitment and in clinic data collection												
Follow up calls at 6 months												
Data entry and cleaning												
Data analysis, write up and dissemination												

In the next sections we will describe the proposed research for 3 individual projects.

Project 1: Understanding and Discouraging Overuse of Potentially Harmful Screening Tests
Project Leader: Stacey Sheridan, MD, MPH

Specific Aims

Most prevention efforts focus on promoting services (e.g. vaccination, screening tests, chemoprevention). While some of these services have clear net benefit, many instead have possible or clear net harm. Currently, three quarters of services graded by the U.S. Preventive Services Task Force (USPSTF) have possible or clear net harm (C, I, and D services). Many of these services are delivered at rates well in excess of what might be expected based on their potential for harm. This leads to adverse outcomes, excess

costs, and missed opportunities to deliver more beneficial care. An important issue in disseminating prevention is how to shift toward a focus on the appropriateness of prevention: encouraging services with clear net benefit and either discouraging or dampening demand for services with possible or clear net harm. Unfortunately, little is known about what drives overuse of potentially harmful screening services or how to make harms salient to patients.

In Project 1, we propose a research agenda that will begin to illuminate an approach to discouraging or dampening services with possible or clear net harm. As part of our agenda, we include both 1) qualitative interviews and 2) a randomized controlled trial (RCT) of 775 patients at 4 primary care practices to assess factors associated with intent to receive possibly or clearly harmful screening services and determine whether and how intent to accept screening changes with various presentations of information about harms (e.g. qualitative, quantitative, narrative, framed). In both the interviews and RCT, we will focus on four exemplar screening services: osteoporosis screening (previous C recommendation and now no recommendation for women <65 years old with no fracture risk factors), PSA screening (I statement for men aged 50-74; D for men 75 and older), electrocardiogram (EKG) and exercise treadmill test (ETT) screening for asymptomatic cardiac disease (D recommendation for men and women at low risk), and colorectal cancer (CRC) screening (C for ages 76-84 and D for 85 and older). Our Specific Aims are enumerated below.

Phase 1 (Year 1): Qualitative Interviews

Aim 1. Determine whether and how individuals conceptualize the harms of preventive screening.

Hypothesis: Most individuals will fail to consider the harms of screening and rely on heuristics when they make decisions about potentially harmful screening.

Phase 2 (Years 2 and 3): Randomized Survey Study

Aim 2. Determine which presentation of harms most effectively reduces intent to accept screening services with possible or clear net harm: a qualitative, quantitative, narrative, or framed presentation. (primary aim)

Hypothesis 1: The combined quantitative, narrative, and framed presentations of harms will reduce intent more than the <u>commonly used</u> qualitative presentation of harm.

Hypothesis 2: The combined narrative and framed presentations of harms will reduce intent more than the <u>commonly recommended</u> quantitative presentations

Hypothesis 3: The narrative presentation of harms will reduce intent more than the framed presentation of harm.

Aim 3. Determine whether the effectiveness of harms presentations in reducing intent to accept screening services with possible or clear net harm varies by exemplar service

Hypothesis: Harms presentations will be most effective in reducing intent for services with low visibility and less overuse (e.g. EKG and ETT screening for asymptomatic coronary disease) and least effective in reducing intent for services with high visibility and more overuse (e.g. prostate cancer screening).

Aim 4. Explore the correlates of intent to accept screening services with possible or clear net harm

Hypothesis: Perceived risk and treatment benefit, self-efficacy, heuristic beliefs, prior screening habits, personality traits, optimism, social norms, and health literacy will all be independently associated with intent to accept screening services with possible or clear net harm.

Significance

Three quarters of USPSTF recommendations for preventive services lead to possible or clear net harm (C, D, I). For these services, the balance of benefits and harms at a population level is alternately 1) too close to call (C services), 2) suggestive of no benefit or net harm (D services), or 3) indeterminate due to lack of sufficient evidence (I services). Whether harm occurs at the individual level depends on the delivery rate of services and, if services are delivered, on the probability of associated adverse events.

Appropriate rates of delivery for C, D, and I services can be inferred from recommendation statements by the USPSTF (www.ahrq.gov) and by ancillary papers supporting USPSTF work (e.g., Sheridan, Harris et al., 2004; Petitti et al., 2009). These sources suggest that for C and I services, appropriate delivery rates might be expected to be around 50% given that the net benefit for an individual depends on personal characteristics and preferences and reasonable people may disagree about the appropriate course of action after shared decision making. On the other hand, for D services where the balance of benefits and harms favors no benefit or net harm, appropriate delivery rates should approach 0%.

Compared with expected delivery rates, potentially harmful screening tests are overused. Although national level data on screening rates for C, D, and I services are sparse, it clear that at least some C, D, and I

services are overused. For instance, 65% of women ages 40-49 have been screened for breast cancer (C recommendation for women ages 40-49) (Coughlin et al., 2004). 75% of men over age 50 have been screened for prostate cancer (I statement for men age 50-74; D recommendation for men older than 75 years) (Sirovich et al., 2003). Additionally, 11% of asymptomatic men and women receive an EKG for screening purposes (D recommendation for men and women at low risk) (Merenstein et al., 2006; Mehrotra et al., 2007).

The factors driving overuse of screening services are largely unexplored. Although many have identified potential sources of screening overuse (such as celebrity or advocacy endorsement (Larson et al., 2005), misreporting of screening services in the media (Katz et al., 2004), or a general culture of prevention (Schwartz et al., 2004), few have systematically explored the impact of such factors. One important national survey about enthusiasm for cancer screening concluded that many individuals feel that screening is an obligation to be continued even if physicians recommend stopping or reducing its frequency and despite the possibility of false-positive test results (Schwartz et al., 2004). However, this survey was not performed in a clinical setting where individuals are most likely to deeply process screening information. Additionally, it explored only a limited number of potential sources of screening overuse.

Potential sources of screening overuse can be inferred from theory and research, but need to be tested. Important potential sources of screening overuse include knowledge deficits, risk misperception, heuristics (i.e. rules of thumb such as "screening is good"), prior screening behaviors, personality traits, health literacy levels, and social norms. Each of these has been linked to screening behavior (O'Connor et al., 2003; Sheridan, Felix et al., 2004; Bogg & Roberts 2004; Larson et al., 2005; Berkman et al., 2011, in press; Ackerson & Preston, 2009; Schwartz et al., 2004). However, only few have been studied in relation to harms and few have been studied in combination, limiting conclusions about their relative importance to screening overuse.

To reduce demand for overused services, strategies for addressing sources of overuse must be explored. One major strategy is to improve the understanding of harms among patients. Other strategies are those targeted at physicians, society, or policy. These strategies are explored elsewhere in this application.

Patient understanding of health information, including harms, is influenced by several factors such as an individual's attention to information, ability to process it, and how and by whom the information is presented. Currently most clinical presentations of harms are qualitative verbal presentations (Kalet et al., 1994; Neuner-Jehle et al., 2011). However, recent research (Berry et al., 2004; Knapp et al., 2004; Covey, 2007) and decision aid standards (Elwyn et al., 2006) suggest that quantitative or numerical presentations (e.g. absolute risk or relative risk with baseline absolute risk) are better understood. Furthermore, other types of presentations may improve the salience of potentially harmful screening tests. Narrative presentations (i.e. presentations that feature a narrator sharing his or her experiences) have been shown to improve understanding and alter intent for behaviors by engaging individuals to process health information. Narratives may also put a human face on screening decisions (reducing individuals' tendency to think bad outcomes won't happen to them) and help individuals identify and clarify their values (Winterbottom et al., 2008). Framed presentations of harms (i.e. those presenting the same information in either a positive or negative light) alter intent for behavior by capitalizing on individuals innate tendencies to seek some risks and avoid others (Salovey & Wegner, 2003; Tversky & Kahneman, 1981).

What this proposal offers: This project (Project 1) offers a research agenda that explores both sources of screening overuse and strategies to increase patients' understanding of harms. This project complements the work of Projects 2 and 3 and the core (described elsewhere in this application), which explore other approaches to reducing overuse of screening services with possible or clear net harm.

Innovation

The proposed work is highly innovative in several respects. First, this work will compare the impact of multiple theory- and evidence-based approaches to communicating harms. Previous work has provided only limited comparisons. Second, this work will be performed at the point of care. This maximizes patients' engagement in decision making and the likelihood that findings will represent decisions that would actually be made if engaged in clinical care. Third, this work examines a spectrum of relevant USPSTF ratings (C, D, and I). This allows the richest possible understanding of factors associated with choice for services with possible or clear net harm. It also provides the largest potential to intervene.

Approach

The Research Team: Stacey L. Sheridan, MD, MPH will be the project leader for this study and is well suited to this role. She has been the PI or a major co-investigator on studies funded by the AHA, NIH, CDC, and AHRQ. She additionally is intimately familiar with both decision-making research and the clinical content area for this application. Relevant to the currently proposed work, Dr. Sheridan convened the Working Group on Shared Decision Making for the U.S. Preventive Services Task Force, is a subcommittee member of the International Panel of Decision Aid Standards, and had led or collaborated in multiple studies investigating the effects of decision aids on heart disease prevention and prostate cancer screening. Cross-cutting this work, Dr. Sheridan has also studied how well patients and providers understand various presentations of risk, the best methods for clarifying patient values, and the effects of literacy and numeracy on health outcomes. Augmenting and complementing Dr. Sheridan's skills are an experienced team of investigators with a substantial history of previous collaboration in research and education. **Russ Harris, MD, MPH** is a clinical epidemiologist and former member of the USPSTF. He was one of the USPSTF leads for recommendations on screening for asymptomatic coronary disease and prostate cancer. He additionally was the PI for a CDC-funded study of informed decision making for prostate cancer screening and an AHRQ funded systematic review on enhancing the quality of colorectal cancer screening. **Noel Brewer, PhD,** is a decision psychologist with research experience in risk perception, use of medical tests, and the effects of false-positive screening tests. **Margaret Gourlay, MD, MPH** is an epidemiologist with a long history of research in osteoporosis screening. **Maihan Vu, DrPH,** is the Director of Formative Research for the Center of Health Promotion and Disease Prevention and has extensive experience in qualitative methods. **Kant Bangdiwala, PhD,** is a senior biostatistician with extensive experience designing, conducting, and analyzing multi-site trials.

Preliminary Studies

The approach Project 1 takes to communicating harms is based, in part, on prior studies performed by the research team. For instance, Dr. Sheridan and colleagues have previously shown in a RCT of 357 patients in one general medicine clinic that absolute and relative risk information presented in the context of baseline risk is more effective at promoting understanding of treatment benefit information than other risk formats (absolute difference in proportion understanding exact treatment benefit: +10-14%, P=0.04) (Sheridan et al., 2003). Additionally, Drs. Harris, Sheridan, and colleagues have shown in a non-randomized controlled trial of 584 men from 3 North Carolina communities that framing PSA screening in the context of more beneficial men's health services improves knowledge about PSA screening compared to presenting information on PSA alone (mean difference in knowledge: +0.8 on 10-point knowledge score, $p<0.05$). (McCormack et al., 2009). Behavioral theory and the published literature supplement this data, showing the potential of these and our other communication approaches for changing intent for screening behavior.

Overview: The proposed work will be conducted in 2 phases. In Phase 1, we will conduct individual interviews with patients to understand whether and how they conceptualize the harms of preventive screening. In Phase 2, we will conduct a RCT of 775 patients from 4 practices in a practice-based research network to 1) assess factors associated with intent to receive possibly or clearly harmful screening tests (e.g. knowledge, heuristics, conscientiousness) and 2) determine how intent changes with various presentations of information about harms. In both phases, we will focus queries on 4 screening services that are overused and represent a spectrum of USPSTF recommendations and potential harms; these are described below. We considered including services that involved immunization or chemoprophylaxis, but felt that these services involved fundamentally different decision making and thus constrained our choice of services to screening tests.

Screening Services for Study

Osteoporosis Screening in Women <65 years old with no risk factors (previous C recommendation and now no recommendation with no new available data) (USPSTF, 2011): No national data are available on osteoporosis screening in young women without risk factors. However, regional data suggest that osteoporosis screening is likely overused despite lack of clear net benefit. In a sample of HMO participants from Northern California, 62% of women who received bone density tests were age 45-64 years old and 45% of those had normal bone density. Potential harms from osteoporosis screening include anxiety (Cummings SR 2006) and the risks of prolonged bisphosphonate use, including osteonecrosis of the jaw (Woo et al., 2006; Khosla et al., 2008), atypical femoral fractures (Odvina, et al., 2010; Odvina et al., 2005; Park-Wyllie et al., 2011), and esophageal cancer (Green et al., 2010).

Prostate screening for men over age 50 (I statement for men age 50-74; D recommendation for men aged 75 and older): Seventy-five percent of U.S. men over age 50 have had a PSA test (Sirovich et al., 2003) despite

conflicting evidence of the benefits of prostate screening (Andriole et al., 2009; Schröder et al., 2009) in the face of well documented harms (Lin, et al., 2008). This makes prostate screening one of the classic examples of screening overuse in the United States. Prostate screening is additionally an excellent example of screening that has been heavily influenced by social norms because prostate screening has been widely promoted by celebrities, advocacy groups, and even postage stamps (Woloshin et al., 1999). Potential harms from prostate cancer screening include complications of biopsy, impotence and incontinence from unneeded diagnosis and treatment, and the psychological harms of false positive screening. (Lin, et al., 2008; Carlsson et al., 2007)

EKG and ETT screening for cardiac disease for men and women at low risk (D recommendation): Eleven percent of average risk U.S. men and women have had EKGs (Merenstein et al., 2006; Mehrotra et al., 2007) despite clear evidence for lack of benefit from these services in the face of known harms (Pignone, 2003). It is unclear how many are screened with ETT, however, programs that provide executive physicals routinely provide such services. Potential harms include anxiety, complications related to additional testing (especially if testing proceeds to cardiac catherization) (Pignone, 2003; Fowler-Brown et al., 2004), and the risks of overtreatment with drugs such as statins and aspirin (Pignone, 2003; Fowler-Brown et al., 2004).

CRC screening for men and women >75 years old (C recommendation for age 76-85, D recommendation > 85 years old): 12% of U.S. men and women over the age of 80 have had colorectal cancer screening (Ananthakrishnan et al., 2007) despite clear evidence of lack of benefit from screening in this age group (USPSTF, 2008). Potential harms include complications related to unneeded colonoscopy, including bleeding, potential bowel perforation, nausea, abdominal pain, and ileus (Warren et al., 2009).

Theoretical Basis: Our study will not be guided by a single theory. Instead, it will test the relevance and impact of concepts drawn from several major communications, behavioral, and psychological theories, which have relevance to understanding and processing information about harms. These theories include the Elaboration Likelihood Model, the Protection Motivation Theory, and Prospect Theory. According to the Elaboration Likelihood Model, individuals develop attitudes and intent for behavior through one of two processes: effortless heuristic processing of information or central effortful processing of information (Petty et al., 2002). Which process is used depends on an individual's ability and motivation to process the information provided. If individuals are unmotivated or unable to process information, they use heuristics such as "prevention is good" or "my doctor is talking to me about this, it must be good." However, if individuals are motivated and able to process information, they engage in a process of weighing information presented. The Protection Motivation Theory provides insight into what factors are assessed during central processing (Neuwirth et al., 2000). This theory suggests that, in situations with potential harm, individuals develop intent to adopt behaviors by assessing both the threat (including the likelihood it will occur and its severity) and their personal ability to cope with that threat (including the efficacy of available options to reduce the threat and their self-efficacy to carry out such options). Prospect Theory provides further insight into decision making in the face of potential harm (Tversky & Kahneman, 1981). This theory suggests that "loss-framing" screening services encourages their use (Salovey & Wegner, 2003). However as we wish to *discourage* use, we will use a gain frame (i.e. emphasizing that not screening is beneficial and screening can cause harms). Prospect theory also suggests that individuals generally focus narrowly on the consequences of decisions and need help with the appropriate reference point (in this case, safer screening options).

Phase 1: In *Phase 1*, we plan to conduct individual interviews to understand whether and how individuals conceptualize the harms of preventive screening. We'll first broadly explore how individuals make decisions about whether or not to accept screening. We'll then probe to understand whether individuals use heuristics and/or cognitive processing of harms or express decision making consistent with loss aversion. We'll also be alert for the expression of personality traits that may affect intent for screening, such as conscientiousness, neuroticism, and optimism.

To conduct individual interviews, we'll collaborate with Maihan Vu, the director of formative research for the Center for Health Promotion and Disease Prevention. With Dr. Vu, we will develop facilitator guides, recruit participants, and conduct the interviews. By design, we will recruit 48 men and women, aged 50-85, from 4 practices in Duke University's practice-based research network. These same practices will supply participants both for the randomized survey in Phase 2 of this project and for the physician pilot described elsewhere in this application. By using practice-based electronic health records, we will purposively sample individuals so that half have been previously screened for the possibly or clearly harmful services of interest to this project (enumerated above). We will additionally purposively sample to ensure that a quarter of participants across sites have been screened for each of our exemplar services. All interviews will be conducted by a trained interviewer, transcribed verbatim, and analyzed using inductive and deductive reasoning with the assistance of

qualitative analysis software. Results will be triangulated with results from the physician pilot (Project 2) and used to guide development of the randomized survey and its vignettes that will be administered in Phase 2.

Phase 2: In *Phase 2*, we plan to conduct a RCT of 775 patients from 4 practices in Duke's Primary Care Research Consortium (PCRC) to 1) assess factors associated with intent to accept possibly or clearly harmful screening tests (e.g. knowledge, beliefs/heuristics, personality traits) and 2) determine whether and how intent changes with various presentations of information about harms. An overview of the study is depicted in the Figure. As is evident, participants will be randomized to 1 of 4 presentations of information about harms (qualitative, quantitative, narrative, or framed) and each presentation will be delivered for up to 4 clinical vignettes (osteoporosis screening in women age 50-64 with no risk factors, PSA screening in men age 50-85, EKG and ETT screening for men and women age 50-85, and CRC screening for men and women age 76-85) as applicable based on age and gender. Pre- and Post-vignette measures will allow us to assess both baseline factors associated with screening intent and how information presentation affects screening intent.

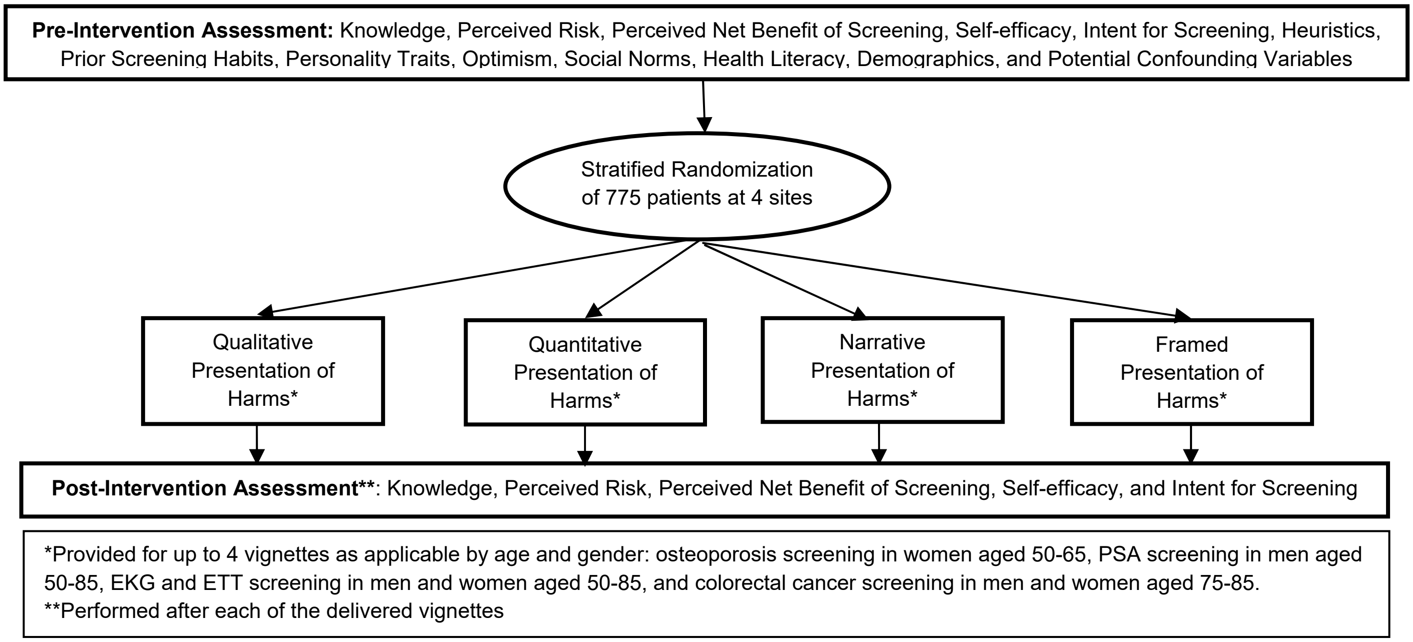

Study Sites: We will recruit study sites from the Duke PCRC (see the UNC-CH Resource Section on page 111 and letter of support on page 165). In brief, the Duke PCRC currently includes 18 family and internal practices that provide routine medical care to adults and will form the source population for this study. From among these practices, we will recruit a convenience sample of four practices for participation in our study. Practices will be included if they are willing to provide access to their patients' medical records for the purpose of pre-screening for enrollment and if they have physicians who are willing to participate in qualitative interviews for triangulation of patient findings. We will exclude practices that the network determines are in turmoil because of turnover or major management problems. Enrollment will not depend on practice type or medical record type. Following the general policy of the Duke PCRC, we have not solicited letters of support from practices. The network discourages pre-solicitation because: a) the network strives to minimize hassles for the practices and finds that practices often consider their letter of support as seriously as they consider actual participation; b) the network has found that letters of support written months before project initiation are not a valid indicator of practices' ability to participate once a project gets underway due to several intervening factors such as provider/staff turnover and changes in the practice structure; and c) the network has a strong track record of practice recruitment and retention, with average rates above 95% for studies of similar magnitude and with similar reimbursement to what is planned in this study (personal communication: Dolor).

Practice Recruitment and Enrollment: We will use the following protocol to recruit and enroll practices in this study: 1) In collaboration with the PCRC director, the study coordinator will select 4 practices and contact them about participation. 2) For each study site selected, the study coordinator will seek an introduction from the PCRC director (our co-investigator, Rowena Dolor), call the designated liaison to the PCRC, describe the study, and assess initial interest. Alternate practices will be used if those selected are unwilling to participate. She will then arrange an on-site visit between key decision makers at the practice (i.e. lead provider, practice

administrator, and/or staff depending on the practice structure) and a physician recruiter (Dr. Sheridan). 3) The physician recruiter will visit the practice to meet with key decision makers and give a brief overview of the study and its mechanics, soliciting participation of both the practice and 4 or more interested providers who will contribute the patient sample for the study. In accordance with the general policy of the PCRC, the physician recruiter will emphasize both the benefits to the practice and the network goal of minimizing hassle for the practice and providers. The recruiter will clearly explain the role of the practice (e.g. provide a room for research, enroll 4 providers and 194 of their patients over 18 months, provide access to the electronic medical records of patients to facilitate recruiting, answer questions, and negotiate the details of participation.

Patient Population: Our study population will include men and women ages 50-85, who receive continuing care at the participating PCRC sites. To provide the broadest generalizability, we will attempt to include a range of participants, representative of patients who are cared for in these primary care practices. To allow an adequate sample for our analyses, we will purposively recruit our sample so that 50% are women and 75% are under age 65. Additionally because individuals with and without prior acceptance of screening services with possible or clear net harm may be driven by different decision making factors, we will purposively recruit individuals such that half have prior screening for the preventive services of interest and half don't. Exclusion criteria for our study include: 1) treatment of psychosis; 2) history of alcohol or substance abuse within the last 2 years; 3) dementia or other severe cognitive dysfunction; 4) serious medical illnesses with a life expectancy of less than 2 years (e.g. metastatic cancer); 5) inability to speak and understand English; 6) blindness; 7) presentation for an acute medical visit; or 8) no phone.

Patient Recruitment and Enrollment: Patients will be recruited from practice lists of appropriately aged individuals who have an upcoming medical visit with their provider and who were previously screened or unscreened for screening tests of interest. These lists will be generated through queries of the electronic health records in use in participating practices. The study coordinator will send a letter and subsequently telephone all potentially eligible patients regarding participation in a study. During the telephone call, the research assistant will assess eligibility with questions probing for relevant history. The research assistant will then ask potential participants to come to a study visit one hour prior to a scheduled provider visit to enroll and participate in the study. All recruitment and enrollment will be tracked in an access database.

Patient Randomization: Participants will be centrally randomized to 1 of 4 presentations of information about harms (qualitative, quantitative, narrative, or framed) using a computerized random number generator. Randomization will be stratified by study site and gender.

Interventions We plan to test the effects of four alternate presentations of harms: qualitative, quantitative, narrative, and framed. These presentations were chosen for their theoretical potential to reduce the likelihood individuals will accept screening services with possible or clear net harm. For each, we will apply best evidence for presentation.

Importantly, each harms presentation will be provided multiple times to any one individual as they view information about each of the exemplar screening services for which they would be eligible based on their age and gender (up to 2 for women, up to 3 for men). The order of information about exemplar services will be randomized within each study participant to avoid bias. All presentations will be in paper format.
In addition to information about harms, each presentation or vignette will include the following information: a description of the disease to be detected, a statement of the USPSTF recommendation for the service, a description of the possible benefits of the service (for C and I services only), a statement of alternatives to screening, and a statement encouraging decision.

Details about how harms will be presented in the different study arms are outlined below:
<u>Qualitative Presentation of Harms:</u> In the qualitative arm, we will describe harms using verbal descriptors, such as rare, unlikely, likely, or very likely. To reduce the variability in people's conceptions of these descriptors, we will choose descriptors that collectively imply a natural order (as demonstrated above) (Berry et al., 2004).
<u>Quantitative Presentation of Harms:</u> In the quantitative arm, we will present harms as absolute risks with accompanying pictographs (i.e. groups of figures shaded to represent harm rates). Compared with other risk formats, absolute risks have been shown to improve understanding for both low and high literacy individuals. As an adjunct to numerical information, pictographs engage individuals to process information, improve understanding, and are persuasive (Galesic et al., 2009; Garcia-Retamero & Galesic, 2009).
<u>Narrative Presentation of Harms:</u> In the narrative arm, we will present harms using patient narratives (i.e. presentations that feature a patient telling their experiences with harms). For each exemplar service, we will present multiple narratives, each representing information about one of the possible harms associated with that

service. Presentation of multiple narratives (rather than a single narrative) has been shown to increase the amount of change in behavioral intent (Betsch, et al., 2011). To address concerns in the literature that characteristics of the narrator independently influence narrative effect (Winterbottom et al., 2008), we will present narratives in paper format with each narrative page formatted so that a banner of culturally diverse age-appropriate pictures are shown at the top and the series of narratives are randomly listed beneath as we have done in other studies.

Framed Presentation of Harms: In the framed arm, we will frame not screening with potentially harmful services as beneficial (i.e. use a gain frame) and compare the potentially harmful services to more beneficial services. By stressing the benefits of A and B recommended services and using wording that emphasizes that A and B recommended services help individuals achieve net health benefit while C, D, and I services do not (or in the case of C and I "do not necessarily help"), we hope to lower interest in C, D, or I services.

Intervention and Survey Administration Procedures: The pre-intervention assessment, interventions, and post-intervention assessment will all be administered prior to one clinic visit in a private room in the clinic. After completing informed consent and their baseline assessment, participants will be randomly assigned to their intervention arm and view the exemplar service vignettes (with harms presented per random assignment) that are applicable to them based on their age and gender. After each vignette, participants will complete relevant follow-up assessments. All surveys and intervention vignettes will be delivered in paper format.

Because accurate implementation of our interventions and surveys is essential for study fidelity, we will provide our study coordinator and research assistants with intensive training on intervention and survey implementation. We will additionally provide weekly monitoring. For these activities, we will rely heavily on study protocols and tracking systems used in our previous studies.

Practice and Participant Retention: To ensure practice retention, we will exclude practices that are in turmoil because of turnover or major management problems; solicit practice-wide support by visiting each participating practice in person to explain the study and provide education to providers and staff; and make all study requests clear and specific. We will additionally provide $1000 to each practice for each year of participation to defray costs for space and staff time in providing clinic schedules.

To encourage complete participant participation, we will offer a moderate monetary incentive ($35). We will additionally schedule their study visit coincident with a regularly scheduled clinic visit, calling participants to remind them of their visits if necessary.

Measurement: Our planned measures are described below and delivered as shown in the flow diagram on page 132. We will perform cognitive testing of these measures on 5-10 patients representative of the variability of our source population prior to full survey administration.

Aims 2 and 3: Determine which presentation of harms most effectively reduces intent to accept screening services with possible or clear net harm

Intent to accept screening: Following the example of others (Betsch et al., 2011; Berry et al., 2004), we will measure intent to accept screening services with possible or clear net harm with a single item "How likely is it that you will have (name of screening test)?" Responses will range from "very unlikely" to "very likely".

Aim 4. Explore the correlates of intent to accept screening services with possible or clear net harm

Knowledge: We will assess participants' knowledge of the potential harms of exemplar screening services by asking them to identify applicable harms from among lists of possible harms as we have done in other knowledge assessments (Sheridan et al., 2006).

Perceived risk: We will focus on risk perception relevant to Protection Motivation Theory and assess perceptions of 1) the severity of each disease to be prevented by exemplar screening services and 2) the probability of contracting those diseases. For each assessment, we will use single item questions with Likert responses (Neuwirth et al., 2000).

Perceived net benefit from screening: In accordance with Protection Motivation Theory, we will evaluate participants' perceived net benefit from each exemplar screening service adapting questions from our prior work (Lewis et al., 2003). Specifically, we will ask individuals to state the likely outcome of screening for each service. Response options will include: "On average, people who are screened are more likely to benefit than be harmed," "On average, people who are screened are equally likely to benefit and be harmed," and "On average, people who are screened are more likely to be harmed than benefit."

Self-efficacy for screening: To assess participants' self-efficacy for carrying out each exemplar screening service, we will ask individuals "How confident are you that you could get a (name of screening test)?" Response options will range from "not at all confident" to "very confident" (Driscoll et al., 2011).

Heuristic Beliefs: As in our prior work (Driscoll et al., 2011), we will measure heuristic beliefs using single items that assess participants' agreement (on a 5-point Likert scale) with common beliefs. Specifically, we will explore agreement with the following statements: "Everyone should have a regular (name of screening test)," "When my doctor discusses a test, I should get it," and "My insurance company pays for recommended screening tests."

Prior Screening Habits: We will measure participants' prior screening habits with single item questions asking about prior receipt of exemplar screening tests. Participants' self-report will be correlated with screening reported in the electronic medical record.

Personality Traits: Personality traits have been previously correlated with prevention (Bogg & Roberts, 2004). We will measure personality traits that we think may be related to acceptance of potentially harmful screening tests (e.g. neuroticism, conscientiousness) using the Ten Item Personality Inventory (Gosling et al., 2003). This score measures conscientiousness using two-items that ask participants to indicate their agreement (on 7-point scale ranging from strongly disagree to strongly agree) with the following statements: "I see myself as dependable, self-disciplined" and "I see myself as disorganized, careless". It measures neuroticism using two-items that ask participants to indicate their agreement with the following statements: "I see myself as anxious, easily upset" and "I see myself as calm, emotionally stable."

Optimisim: We will measure optimism using the Optimism Subscale of the Life Orientation Test (alpha 0.87) (Scheier et al., 1994). This subscale asks participants to indicate their agreement (on a 5-point scale ranging from strongly disagree to strongly agree) with 3-items: 1) "In uncertain times, I usually expect the best," 2) "I'm always optimistic about my future," and 3) "Overall, I expect more good things to happen to me than bad." Scores on this subscale have been shown to correlate with health behavior.

Social Norms: Multiple forms of social pressure may affect screening. We will ask participants about the social pressure exerted by providers, families, and the media and about their motivation to comply with these sources of pressure using 6-items adopted from Fishbein et al. (2001).

Health Literacy: We will assess health literacy using measures of both reading skill and numeracy. We will assess reading scale using the well-validated Rapid Estimate of Adult Literacy in Medicine (REALM) measure (Davis et al., 1991). We will assess numeracy using the well-validated 3-item scale from Schwartz and Woloshin. (Schwartz et al., 1997) Both of these measures have been correlated with health outcomes.

Other measures/potential confounders: We will measure the following variables as potential confounders: age, socioeconomic status, insurance status, race, family history of disease, and perceived general health status. We will also measure process variables, including patients' perceptions that vignettes were credible.

Data Capture/Management/Quality Assurance: The research team will use TELEform® surveys to facilitate collection, entry, and export of data. TELEform® software uses optically scannable data collection instruments for data capture, thus eliminating the need for double data entry; it also reduces data editing since range checks are built into all choice option fields and data verification is done on-screen. To ensure high quality data, we will prepare documentation protocols for all data collection instruments and procedures. Project staff will carefully edit all paper surveys and submit completed surveys for data capture and verification. Trained personnel will scan the data from the hard copy scannable surveys into computer files. Raw data on survey paper forms will be kept in locked files, in locked offices at the University. Participant names will be kept separate from data (i.e. no reports will link individual names with data). All electronic data will be maintained on secure servers and will undergo frequent and regular back-ups. Statistical staff will perform range checks, consistency checks (overall and by practice), and other procedures to ensure data quality. Data will be cleaned and frequency distributions and univariate descriptive statistics will be computed for all study variables in order to identify outliers and possible influential observations. Distribution anomalies may be resolved through variable transformation or utilization of robust nonparametric methods for analysis.

Analysis

Aim 2. Determine which presentation of harms most effectively reduces intent to accept screening services with possible or clear net harm: the qualitative, quantitative, narrative, or framed presentation (primary aim)

For our primary analysis, we will proceed in 4 steps. First, we will perform an omnibus F test to detect any mean difference in intent to accept screening services with possible or clear net harm at an alpha level of 0.05. Second, we will compare the combined effect of the quantitative, narrative, and framed presentations of harms (versus the qualitative presentation) on intent to accept screening services. Third, we will compare the combined effect of the narrative and framed presentations of harms (versus the quantitative presentation) on

intent. Finally, we compare the effect of the narrative versus framed presentations on intent to accept potentially harmful therapy. For all comparisons, we will use a Generalized Linear Mixed Model (GLMM) to account for clustering of screening services within an individual (for example, a male age 60 will have two data points for intent, one for PSA screening and one for asymptomatic cardiac screening). The GLMM will include each patient as a random effect and baseline intent for screening and practice site as fixed effects.

Analyses are described assuming that randomization will result in an equal distribution of patient characteristics between the intervention and control groups. If necessary, the intention-to-treat analyses will control for baseline variables that were not similar among groups. All data analysis will be performed using SAS (Cary, NC) software and will account for multiple comparisons (see sample size section for details). Because our study occurs at one point in time, we expect minimal attrition; nevertheless, some drop-out is possible. We will record the reasons for not completing the follow-up interviews and use this data to help understand whether the corresponding data are missing at random (MAR) or whether missingness is informative. If missingness is non-informative, we will consider imputing endpoint data using multiple imputation techniques (Little & Rubin, 1997).

Aim 3. Determine whether the effectiveness of harms presentations in reducing intent to accept screening services with possible or clear net harm varies by exemplar service

To determine whether intent to accept potentially harmful screening services varies based on exemplar services, we will perform subgroup analyses. In these analyses, we will focus individually on each exemplar service using simple (adjusting only for baseline intent and practice site) and multivariate regression models, instead of GLMMs, because within exemplar services each person has a single intent measure. We have powered our subgroup analyses to have a sufficient sample size for at least two exemplar services (asymptomatic cardiac screening and PSA screening) that are applicable to all participants or all participants of one gender. The analysis of the other two services will be considered exploratory given they are applicable to only smaller subsets of participants.

Aim 4. Explore the correlates of intent to accept screening services with possible or clear net harm

To determine the correlates of intent to accept potentially harmful screening services, we will first examine the bivariate relationship between several potential correlates (e.g. knowledge, risk perception, heuristics, prior screening habits, personality traits, and health literacy) and intent to accept potentially harmful screening services using bivariate t-tests adjusted for patient level clustering (simple GLMMs). We will then enter variables that have a bivariate p value of less than 0.2 into GLMMs to determine the independent effect of those variables. For each variable significantly related to intent to accept screening, we will report percent variance; this will allow investigators to determine how to best target future messages on harms.

Sample size: We based our sample size estimates on the minimal clinically important difference in intent to accept therapy: a 0.5 point difference on a 5-point scale. A study of a prostate cancer screening decision aid that showed this difference in intent (on a 3-point scale), also showed a 21% absolute reduction in PSA screening tests completed (Volk et al., 2003). Published studies comparing alternate presentations of harms suggest that we will be able to detect such differences between presentations we have chosen. For instance, a study comparing quantitative to qualitative presentations of medicine side effects showed an absolute mean difference in intent to accept therapy of 1.1 (on a 6-point scale; equivalent to 0.9 on 5-point scale) (Berry et al., 2004). Furthermore, a study comparing narrative to quantitative presentations of vaccine risks showed an absolute mean difference in intent to accept therapy of up to 1.3 (on 7-point scale; equivalent to up to 0.9 on 5-point scale), depending on the number and quantity of narratives (Betsch et al., 2011).

Because we plan to compare the effects of harms presentations in both the overall sample (aim 2) and in subgroups of exemplar services (aim 3), we have calculated our sample sizes with attention to both the overall and subgroup levels. We conservatively base sample size estimates on two-sided t-tests with an alpha level of 0.001 to test our three hypotheses in the overall sample. We assumed a standard deviation of change to be 1 based on data from the literature (Betsch et al., 2011) and a conservative expected correlation of 0.75 between pre-post measures. We additionally assumed a large intra-class correlation (0.8) for the clustering of intent to accept screening tests within individual patients answering questions about multiple screening services. The cluster size is on average 2. With these assumptions, we will need 184 participants in each of 4 intervention arms to have approximately 95% power to detect a 0.5 point difference in mean changes in intent to accept screening services between the any of the active intervention arms and the qualitative presentation control (Liu & Wu, 2005; Donner & Klar, 1996; Donner & Klar, 2000). We chose this overall sample size of 184/intervention arm because it provides approximately 80% power to detect a 0.5 point mean change in intent to accept screening for the subgroup analyses of screening for asymptomatic cardiac disease and prostate

cancer. Table 4 below shows the sample sizes, power estimates, and alpha levels for subgroup analyses pertinent to individual screening services (aim 3) based on an overall sample size of 184 patients. The overall alpha (type 1 error rate) will be 0.05 (□=0.001*3+ 0.001*3 +0.015*3+=0.051).

Table 4

Screening Service	No. of Participants in Each Study Arm	α Level for each Comparison	α Level for All Tests	Actual Power (1-β)
EKG	184	0.001	0.003	0.80
PSA	92	0.015	0.045	0.82
Osteoporosis	73	0.05	-	0.85
Colonoscopy	47	0.05	-	0.67

We expect attrition and plan to enroll 775 patients (184 +5% attrition (=194) in each of 4 intervention arms).

Timeline: For a timeline of project 3, please see Table 3 on page 127.

Potential Limitations and Contingencies: As is true of any study, this study has potential limitations. These include: 1) Inability to maintain an adequate practice pool. To avoid this, we have planned generous reimbursements for practices and minimized burden on the practice. We will additionally recruit as many providers per practice as possible to allow for attrition. If a provider or practices chooses to end participation, we will recruit additional practices and providers from within the Duke PCRC. 2) Inability to maintain adequate participant pool. To avoid this, we will reduce the burden of participation as much as possible, schedule visits coincident with MD visits if possible, and offer a generous incentive for participation.

Future Steps: Findings from our study will fuel future research, which might include 1) testing of additional communication strategies or 2) incorporation of successful communication strategies into a either a harms communication toolkit or individual decision aids for further evaluation.

Project 2: Understanding Physicians' and Patients' Views of Harms and Clinical Preventive Services
Project Leader: Maihan Vu, DrPH, MPH

Specific Aims

Three quarters of U.S. Preventive Services Task Force (USPSTF) recommendations for preventive services are associated with possible or clear net harm (C, D, I) and many of these services are delivered at excessive rates. While physicians play a direct role in deciding what services are appropriate for their patients, how they make screening decisions or the factors that influence decisions about potentially harmful services has not been well studied. Prior studies have highlighted a number of factors that affect adherence to practice guidelines for clearly beneficial services, but few have examined how physicians use recommendations about services with potential net harm. To better understand how physicians make these decisions, it is important to examine the factors at multiple levels that may shape their views of potentially harmful preventive services.

The objective of this formative study is to understand physicians' knowledge, attitudes and experiences related to making decisions about the use of screening and preventive services that have possible or clear net harm (C, D, and I services). We will employ an innovative combination of qualitative and quantitative methods to obtain an in-depth profile of perceived harm and factors that affect making evidence-based decisions about four exemplar screening services: osteoporosis screening for women ages 50 to 64 with no fracture risk factors; prostate cancer screening for men ages 50 to 85; electrocardiogram (EKG) or exercise treadmill testing (ETT) screening for women or men ages 50 to 85; and colorectal cancer screening (CRC) for women and men ages 70 to 85. Our two phase approach begins with surveys and semi-structured interviews with primary care physicians to explore both the depth and breadth of physicians' beliefs and attitudes toward harms of preventive screening and how they make decisions about use. In the second phase, we will create case studies of selected patients and their physicians from Project 3 to describe the context and influences for making decisions about CRC screening. These case studies will result in an in-depth analysis of how decisions are made, linking findings from Project 1 and Project 3 both described elsewhere in this application.

Phase 1 – Physician Phase
Aim 1: Determine whether and how physicians conceptualize the harms of C, D, and I USPSTF-rated services.
Phase 2 – Case Study Phase

Aim 2: Describe the context and influences for patients' and physicians' decisions about potentially harmful clinical preventive services.

Significance

How physicians approach screening decisions is not clear. Physicians make decisions every day about whether to screen patients for a variety of potential health problems. To make these decisions, physicians must engage with evidence that suggests wide variability in the utility and safety of screening protocols. While evidence-based guidelines (such as the Guide to Clinical Preventive Services) exist to guide safe and cost effective approaches to screening for many health conditions, numerous studies suggest that often these recommendations are not followed. Of particular concern are screening tests that have the potential to cause harm through false positives, anxiety, labeling, or overdiagnosis. Little is known about the physicians' beliefs and decisions about screening tests that may cause harm. However, some evidence suggests that physicians generally favor screening and overuse services with possible or clear net harm (Lawson et al., 2005, Katz et al., 2004, Schwartz et al., 2004).

Factors influencing overuse of screening services need to be further explored. Previous studies have identified potential factors that might lead to screening overuse including lack of awareness or familiarity with current guidelines, lack of agreement with guidelines, and lack of self-efficacy for counseling on screening (Cabana 1999, Krist 2007). Other studies have noted that despite appropriate knowledge and skills, a physician's ability to carry out screening recommendations may also be limited by external factors including patient or environmental factors (Guerra, Jacobs, 2007). However, few have studied how the combination of these different factors may influence physicians' views and screening practices for services with possible or clear net harm.

What this proposal offers. This project provides an important first step to increase our understanding of how physicians and patients view the potential benefits and harms associated with four exemplar screening services. This research examines both physician and patient perspectives to address: 1) how they think about the potential harms of these services 2) whether and how they view harms in relation to benefits in making decisions about screening, and 3) how they use these understandings to make decisions about use of services. This research also addresses the contextual conditions within which decisions are made. The integration of a variety of data sources to further explore decision making ensures that the issues are not explored through one lens, but rather a variety of lenses which allows for multiple perspectives to be revealed and understood.

Innovation

The major innovations of this proposal include: 1) a comprehensive formative evaluation triangulating methodologies, data sources, and respondents to gain deep and broad insight into how decisions are made about four potentially harmful preventive services and 2) a multidisciplinary team of experienced collaborators who will use the formative evaluation in an integrated way, sharing data to provide a more holistic understanding to help develop interventions that are tailored to the physicians and patients in the practice setting.

Approach

The Research Team. The research team, led by **Maihan B. Vu, Dr.PH, MPH**, has extensive experience with qualitative research methods. Dr. Vu, the Director of Formative Research for the UNC Center for Health Promotion and Disease Prevention, has provided qualitative leadership on numerous NIH and CDC studies. Relevant to this current proposal, Dr. Vu is collaborating on formative and evaluation components of several clinical practice and community based intervention research studies including practice engagement, patient recruitment, training and conducting interviews, data analysis, interpretations and application. Key studies include the Kids Eating Smart and Moving More Study, an NICHD funded pediatric obesity intervention study conducted with 24 primary care practices serving Medicaid families throughout the state of North Carolina and Heart Healthy Lenoir, an NHLBI Health Disparities Center grant including an integrated set of three interdisciplinary studies to create innovative, evidence-based and community-based strategies to reduce cardiovascular disease in Lenoir County, NC, a rural, economically distressed region. The co-investigators on this team have significant overlapping experiences, including qualitative data collection with physicians and patients on a broad range of clinical preventive health care services and national expertise in medical decision making. **Stacey Sheridan, MD, MPH,** a physician and associate professor of medicine (PI for Project 1), has extensive clinical and decision making research expertise. Drs. Sheridan and Vu have collaborated in

education and on multiple qualitative studies including a comparative effectiveness study and the Heart to Health Study to examine individuals' responses to global CHD risk. Dr. Sheridan's research identifies several influential factors in decision-making and highlights the importance of patient participation in decision-making for both CHD prevention (Sheridan et al., 2009) and prostate cancer screening (Sheridan et al., 2004). **Carmen Lewis, MD, MPH,** (project leader for Project 3) has conducted qualitative research, with a focus on both physicians and older patients regarding colon cancer screening. Importantly, Dr. Lewis' research provides insights on how physicians incorporate information when making recommendations about colon cancer screening for older adults (Lewis et al., 23009, Lewis et al., 2008), representing one of our target screening services for this application. **Noel Brewer, PhD,** is an expert in medical decision making with an emphasis on risk communication. Drs. Brewer and Vu have previous educational collaborations. Dr. Brewer's experience in qualitative methods include leading an eight university collaboration to cognitively test HPV vaccine survey items, and co-leading a qualitative project to assess high risk women's thoughts about HPV DNA self-test collection devices. He has led several dozen quantitative survey studies.
In summary, we have an experienced research team blending strong research skills with extensive primary care and public health savvy. In addition, this proposal promises a rich convergence of qualitative and quantitative expertise.

Study Site. Phase 1 interviews and surveys will be conducted with physicians from practices in the Duke Primary Care Research Consortium (PCRC). For a detailed description of this network and letter of support, see Resource Section (page 111)**.** In brief, the Duke PCRC currently includes 18 family medicine and internal medicine practices serving adults. From among these practices, the PCRC Director (a study collaborator) will assist in recruiting a convenience sample of four practices for participation in our study. Because these four practices will also be sampled for Project 1, practices will be included if they are willing to provide access to their patients' medical records for the purpose of pre-screening for enrollment and if they have physicians who are willing to participate in qualitative interviews for triangulation of patient findings. For Phase 2, we will broaden our sample to the 10 practices participating in Project 3. This will result in over 40 physicians, and approximately 10 patients per physician. From this sample, we will select four patients who are at high risk of experiencing net harms from screening and their physicians. See Project 3 for full practice and patient recruitment methods.

Data Collection Phase I – Physician Phase (Year 1)

During this phase, we will use a multi-method approach to assess factors relevant for physicians in making decisions about the use of four exemplar screening services (osteoporosis screening, prostate cancer screening, EKG or ETT screening, colorectal cancer screening) that may cause harm. We will then use this information in triangulation with other data to help inform the development of intervention components to be implemented in Project 1 and the interpretation of findings in Project 3.
Aim 1: Determine whether and how physicians conceptualize the harms of C, D, and I USPSTF-rated services.
Qualitative Interviews. We will conduct eight individual interviews with physicians from four practices in the PCRC. Patients of these physicians will be participants in the interview and randomized trial studies in Project 1. Two physicians at each practice will be invited to participate in the Project 2 interviews. To get a diverse range of perceptions, we will purposefully recruit the lead physician and a junior physician (i.e., joined the practice in the last 5 years) to participate in the interviews. We will recruit physicians as we visit practices to discuss the project and solicit their participation (e.g., at practice meetings). Each interview will last 45-60 minutes. Structured, in-depth interview questions will assess physicians' knowledge and perceptions of potential harms associated with the four clinical preventive services and issues related to how they weigh risks of these services, and the trade-offs between benefits and harms in making decisions to recommend screening. We will also ask physicians about the factors they believe are important to consider in making such decisions. Through interviews, we will generate preliminary information that can be used to understand the depth of the factors that influence making decisions about use of potentially harmful screening services. All interviews will be conducted by a trained interviewer, transcribed verbatim, and analyzed using inductive and deductive reasoning with the assistance of qualitative analysis software. Results will be triangulated with results from patient interviews in Project 2. Physicians will receive $100 compensation for their interviews.
Quantitative Survey. To complement our qualitative assessment, we will conduct a survey with physicians about their knowledge and attitudes towards potential harms of the four exemplar screening services, and their use of harms in screening decisions. All PCRC physicians who provide adult primary care (approximately 100 physicians) will be invited to participate. This initial survey will be developed based on formative work with physicians and conducted at the start of Year 1 and again a year later. Repeating the survey will allow us to

identify changes over time due either to secular trends or the effect of participating in our study. The brief surveys will address physicians' knowledge of potential harms of the screening services, perceptions of the magnitude of those harms, how these perceptions affect their decision-making with patients, and how their decision making is affected also by such factors as medicolegal concerns, clinical cultural norms, and perceptions of what different patients want/would understand. Surveys will be delivered to all practices, with prepaid stamped envelopes for returning them. Physicians will receive $10 compensation for each completed survey, a typical amount for the PCRC to effectively recruit physicians to complete surveys. See Project 1 for full recruitment and enrollment methods.

Data Collection Phase 2 – Case Study Phase (Year 2-3)

To achieve a richer understanding of the context and influences regarding decision making about the use of exemplar screening services, we will develop case studies to generate physician and patient profiles. The case studies will build on data generated from the physician phase in this project with data collected from patients in Project 1 to provide deeper understanding of the context of clinical decisions. They will also facilitate interpretation of the results in the larger randomized controlled trial (Project 3).

Aim 2: Describe the context and influences for patients' and physicians' decisions about potentially harmful clinical preventive services.

In order to describe the context and influences for physicians and patients in making decisions about CRC screening in older people with co-morbidities, we will create four real case studies. Of the 400 participants who partake in Project 3, we will select four patients who are at high risk of experiencing net harms from screening and their physicians to gather comprehensive, systematic, and in-depth information on perceptions of harm and their decision making process. The focus of this aim is to better understand decision making that results in overuse of CRC screening and over diagnosis of CRC. As such we will select patients with significant co-morbidities (Charlson Comorbidity Level 4 or greater) who decide in favor of screening and/or complete CRC screening tests and report discussions with their providers about CRC screening. Then we will select their physician as a complementary case. Each case study will begin with a synthesis of existing data sources; include all of the quantitative and qualitative information collected from Projects 1-3. The information and findings generated from Projects 1 and 2 will be directly incorporated into the in-depth questions asked of these selected patients. Key areas include how patients and physicians view harm, how they might use information about potential harm, and ultimately what is most influential in their decisions about screening use. The case studies will present a holistic portrayal to help us understand the multi-level factors that influence how patients make decisions about use of clinical services.

Analysis

Aim 1: Determine whether and how physicians conceptualize the harms of C, D, and I USPSTF-rated services.
Aim 2: Describe the context and influences for patients' and physicians' decisions about potentially harmful clinical preventive services.

For our qualitative analysis of physician interviews in Phase 1 and patient interviews in Phase 2, we will transcribe each interview verbatim and each transcribed file will be given a unique electronic name. ATLAS.ti, 6.2, a qualitative data analyses software program, will facilitate the analysis. Members of the research team will develop a codebook based on a deductive and inductive process (Miles & Huberman, 1994; Ulin et al., 2005, Strauss & Corbin, 1990). Prior to data collection, a list of codes will be created from the research questions and key variables. Two independent researchers will work together to reach consensus on coding. The initial data will be collected, written up and reviewed. Beside the responses, categories will be generated and a list of themes will be developed. After each interview is coded, text retrievals on specific codes or combination of codes will be completed. These retrievals enable content analysis of particular topics for identification of similarities and differences in themes by respondents (i.e. Provider 1 vs. Provider 2). During analysis, the research team will assess levels of agreement and salience of themes. All qualitative data analysis will be directed by Dr. Vu at the UNC Qualitative Research Unit (QRU).

For the quantitative surveys with physicians in Phase 1, we compute frequency distributions and descriptive statistics for all study variables. Because the surveys occurs at two points in time (Year 1 and Year 2), we will compare response rates to assess whether or how physicians' knowledge and perceptions of potential harms of screening services change over time. The quantitative data analyses will be conducted using SAS (Cary, NC) software and managed by ReCPS Core Office Biostatistics Lead, Dr. Bangdiwala.

To protect participant privacy in this project, names will not appear on interview notes, audio recordings, or surveys. ID numbers will be used for participants and the key that links name, ID number, and

the study data we collect will be stored in a locked file cabinet and/or password protected computer. All study data, including audio recordings will be destroyed when the project is complete.

Issues in Qualitative Research Sample Size. With qualitative data, individual cases make a difference. Outliers and small sample sizes offer valuable information about a particular topic. Sample sizes depend on what researchers want to know, the purpose of the inquiry, what will be useful, what will be credible, and what can be done with the available time and resources (Patton, 1990). In-depth information from a small number of participants can be extremely valuable when the interviews are information-rich as may be the case in this formative study. The logic and power of purposeful sampling lies in selecting information-rich cases for study in-depth (Patton, 1990). Purposeful sampling illustrates characteristics of particular subgroups and facilitates comparisons across groups. Because we are interested in examining differences in perceptions by physicians, we will stratify our sample by professional experiences to ensure a diverse range of views on potential harm.

Timeline: For a timeline of project 3, please see Table 3 on page 127.

Significance and Future Directions

To design effective strategies for improving decision making for C, D, and I preventive services, we must understand how potential harms are understood by both physicians and patients, and how that understanding affects decisions about use of these services. A major strength of this proposed work is the focus on understanding how these decisions are made, a naturally occurring event in a natural setting. The use of innovative strategies allows us to obtain a strong handle on the "real life" experience for physicians and patients. Our data collection places emphasis on specific cases embedded in their own context (i.e., practice settings). As a result, we are able to take into account the local context which makes it possible for understanding different levels of influences. Furthermore, the fact that our mixed method data collection occur over a sustained period (i.e., before, during and after the interventions) makes it possible to study not just what the actual factors are that influence decision making, but how and why they play out in a particular setting over time. Our extensive combination of both qualitative and quantitative methods provides a richness and wholeness to reveal the complexity of understanding decision making of physicians and patients in the real context of the practice environment. This completeness of information will provide our research team and others with the data needed to develop strategies that can reduce potential harms for patients.

Project 3: Improving Appropriate Colorectal Cancer Screening in Elderly Patients
Project Leader: Carmen Lewis, MD, MPH

Specific Aims

The United States Preventive Services Task Force recommends against *routine* colorectal cancer (CRC) screening in patients ages 76 to 85. The goal of these recommendations is to achieve appropriate screening by targeting the healthiest patients with the longest life expectancy and avoiding screening in those with multiple co-morbidities, who are likely to experience net harm. This individualized approach has the potential to improve the safety of CRC screening in the elderly; however, evidence demonstrates a gap between these recommendations and actual clinical care. An important barrier to achieving appropriate CRC screening is the public's unrealistic beliefs regarding the universal benefit of cancer screening and their preferences in favor of screening. In our preliminary studies we developed and tested a decision support intervention targeted to patients 70 to 85 that improved patient knowledge about the harms and benefits of screening, helped patients to clarify their values, and changed patients' screening preferences. Although the initial testing is promising, elderly patients rely on their providers to help guide their CRC screening decisions. Therefore, we propose a randomized controlled trial at the patient level to determine the efficacy of the intervention within a clinical setting. We hypothesize that the use of the intervention will prepare patients for individualized decision making with their providers and result in an improvement in appropriate CRC screening decisions and screening outcomes. To assess appropriate CRC screening decisions and screening, we will use a classification scheme derived from the literature based on age and the Charlson Comorbidity Index. Using this scheme, appropriate screening will include screening for those in the best health because they are likely to benefit, no screening for those in the worst health because they are unlikely to benefit, and evidence of a discussion about CRC screening for those in the intermediate health group because the benefit is unclear.

Aim 1) To examine, in a randomized trial, the effect of a patient decision support intervention on appropriate screening decisions in patients ages 70 to 85 immediately after the index visit with their primary care providers.

The intervention group will have a higher proportion of patients in which the decision about CRC screening is classified as appropriate. Specifically, the proportion of intervention patients in the worst health group who plan to be screened will be lower while the proportion of intervention patients in the best health group who plan to be screened will be higher than analogous patients in the control group. For the intermediate health group, intervention patients will report more discussions regarding CRC screening than control patients after the index visit.

Aim 2) To test the effect of a patient decision support intervention on appropriate CRC screening six months after the index visit with their providers.

Because screening uptake at six months may differ significantly from screening decision outcomes, we will assess screening test completion. Patients in the intervention arm will report a higher proportion of CRC screening classified as appropriate. Specifically, intervention patients in the worst health group will have lower screening rates while intervention patients in the best health group will have higher screening rates than analogous patients in the control group. For the intermediate health group, intervention patients will report more discussions regarding CRC screening than control patients after the index visit.

Aim 3) To examine the extent to which patient decision making factors mediate the impact of the intervention on appropriate CRC decisions and screening.

We hypothesize that the intervention will have more effect when patients are prepared for individualized decision making (demonstrate adequate knowledge and clear values) and when patients' decisional balance and screening preferences align with their particular classification for appropriate screening given their age and health state. We also hypothesize that discussions about CRC screening with providers are necessary for the intervention to be efficacious.

The proposed research will directly address patient safety and the USPSTF recommendations by attempting to target screening in those most likely to benefit and avoiding screening in those least likely to benefit. It will provide new knowledge on how elderly patients perceive the harms and benefits of CRC screening and whether a decision support intervention designed to increase understanding of these tradeoffs can change these perceptions. Finally, if successful in changing patient screening behavior to appropriate screening, the results have broad implications beyond CRC screening in terms of resource use and public trust in the health care system.

Significance

Guidelines for CRC screening endorse individualized decision making to improve patient safety

In 2008, the U.S. Preventive Services Task Force recommended that persons aged 76 years and older not undergo *routine* CRC screening, indicating that the potential to benefit from screening should be considered at an individual level (USPSTF, 2008) Other expert groups, including the American Cancer Society, and the American Geriatrics Society, have made similar recommendations (AGS, 2003; Levin et al., 2008). Taken together, guidelines suggest that decision making about whether or not to undergo CRC screening be individualized based on: 1) a clinical assessment of patients' health status and likely longevity by the provider; and 2) individualized decision making process between the patient and provider where the harms and benefits of CRC are considered in the context of patient preferences about screening. Unfortunately, the recent controversy surrounding mammography screening (Quanstrum, 2010) indicates that most members of the public are unprepared to participate in individualized decision making (Lewis, 2006; Lewis, 2010; Squiers et al., 2011) Research must address this important barrier to individualized decision making if the Task Force Recommendations aimed to improve cancer screening safety are to be realized.

Inappropriate CRC screening decisions put vulnerable patients at risk of harms

Available evidence suggests that the quality of individualized decision making for cancer screening in the elderly is sub-optimal (Walter et al., 2009; Braithwaite et al., 2009; Eddy, 1990). Studies examining cancer screening in elderly people demonstrate both overuse and under use (Gulitz et al., 1998; Rimer et al., 1992; Roetzheim et al., 1995; Rimer, 1993; Weinberger et al., 1991; Caplan & Haynes, 1996; Fox et al., 1997; Burack et al., 1998; Heflin et al., 2002; Sultan et al., 2006; Sima et al., 2010; Holden et al., 2010; Lin et al., 2006; Schonberg, 2008) For CRC screening in particular, older vulnerable patients are subjected to significant potential harms including colonic perforation, bleeding, and even death (Pignone et al., 2002). Older patients, particularly those with poor health status, appear to be at higher risk of complications from colonoscopy

(Warren et al., 2009); furthermore, once they are screened they are subjected to repeated surveillance colonoscopies that may not be indicated (Goodwin et al., 2011). Finally, older adults with poor health status may be diagnosed with a cancer that would never cause them problems (Walter et al., 2005) or they may not be able to tolerate the subsequent treatment necessary to cure them of cancer (Shack et al., 2010; Sunouchi et al., 2000; Sarfati et al., 2009). Conversely, many healthy older adults who are at increased risk of CRC because of advancing age do not receive CRC screening, and as a result, risk death from a preventable cause (Holden et al., 2010). *Clearly, a significant body of research is now available to describe the problem of inappropriate use. It is now critical to develop and test interventions, such as the one proposed in this application, to try to improve appropriate use and patient safety*.

Providers endorse individualized decision making but barriers exist in clinical practice

Individualized decision making could improve appropriate screening if those most likely to benefit are targeted for screening and patients who are not targeted understand that for them, the potential harms likely outweigh the benefits. Our work and others have found that physicians are generally able to make clinical assessments that target screening to those most likely to benefit (Lewis et al., 2008; Cooper et al., 1997; Kahi, 2009; See Preliminary Research). However, there may be significant barriers for physicians to pursue discussions of CRC screening with elderly patients to help them understand why they should not undergo screening. Our qualitative and quantitative work with physicians indicates that while physicians are willing to have brief discussions or provide a simple recommendation in favor or against screening, (Lewis et al., 2009), they may avoid discussions about CRC screening if they think patients may not understand the issues or if the discussions are likely to be lengthy. Others have found that for controversial screening tests (PSA and mammography in women in their 40s) the complexity of the topic and lack of time (Rimmer et al., 1991) are significant barriers to discussions. *Efforts to reduce the burden of these discussions on physicians by having patients prepared for individualized decision could lead to an increase in the number of discussions about CRC screening, improve the decisions about who should get screened, and could result in an improvement in appropriate screening use.*

Elderly patients are unprepared for individualized decision making

Unfortunately evidence suggests that patients are currently poorly prepared for individualized decision making. The recent controversy surrounding mammography screening highlights the consumers' strong belief in cancer screening and their lack of awareness regarding the potential harms (Quanstrum, 2010) Although research is this area has been limited, our work and others demonstrate the public's limited understanding of the tradeoffs necessary in deciding about cancer screening (Lewis et al., 2010; Squiers et al., 2011; Schwartz et al., 2004; Woloshin & Schwartz, 2010) and suggests that while elderly consumers strongly believe in the benefits they lack awareness of the need to make individualized decisions in regards to increasing age and co-morbidity (Lewis et al., 2006; Kistler et al., 2006). *To improve appropriate screening, patients need to be prepared for their role in individualized decisions about CRC screening. Specifically, they need assistance in understanding the tradeoffs between the harms and benefits of CRC screening with age and co-morbidity, assistance in clarifying their values about screening, and help from providers in determining whether or not they will likely benefit from screening.*

A patient decision support intervention can facilitate individualized decision making

Decision support interventions have been shown to facilitate patient understanding of tradeoffs inherent in clinical decision making. A meta-analysis of 55 randomized controlled trials found that they increased decision specific knowledge, produced more realistic expectations for users and reduced decision uncertainty. Further, users were more likely to prefer an active role in clinical decision making, and often opted for more conservative approaches (O' Connor et al., 2009). Importantly for this application, patient decision support interventions for CRC screening have been shown to both increase CRC screening or intent in adults ages 50-75 (6 to 14%) (Pignone et al., 2000; Ruffin et al., 2007; Schroy et al., 2011; Pignone et al., 2011) and decrease PSA screening (22%) (Evans et al., 2005; Volk et al., 2007). Importantly, to achieve appropriate screening we will need to demonstrate both an increase in screening uptake in to those most likely to benefit and a decrease in screening test completion in those most likely to be harmed. While prior decision support interventions for CRC screening have been developed to promote decision making among different screening test options (Pignone et al., 2000; Ruffin et al., 2007; Schroy et al., 2011; Dolan & Frisina, 2002; Wolf & Becker, 1996), they have not been developed to promote individualized decision making in the elderly in regards to whether or not to undergo screening. Although PSA decision support interventions are designed to help men decide about whether or not to undergo screening (Evans et al., 2005; Volk et al., 2007) they have not been designed

specifically to encourage individualized decision making based on health state and age. One decision support intervention targeting women age 70 and older about mammography screening (Mathieu et al., 2007) increased preparation for individualized decision making, but did not change self-reported screening test completion at one month. They did not evaluate appropriate screening in regards to health state. *We have developed a decision support intervention that can address the needs of individualized decision making for CRC screening. Although the initial testing is promising in regards to patient decision making outcomes, a rigorous trial in a clinical setting is needed to determine whether patient decision support can improve appropriate screening decisions with providers and result in appropriate CRC screening behavior.*

Conceptual model (Figure 1): Although the importance of individualized decision making (Fraenkel & Fried, 2010) and a framework for CRC screening has been described (Walter & Covinsky, 2001) in the clinical literature, a theoretical foundation for this work is currently lacking. Therefore, we propose a conceptual model that is based in decision making theory and on a clinical decision making model proposed independently by both Eddy and Deber (Eddy, 1990; Eddy, 1990; Deber et al., 1996). Key constructs include decisional balance as a motivator for patient decision making (Janis & Mann, 1977) and the provider's clinical assessment of benefit to assist patient decision making. We propose that the decision support intervention increases awareness for the need for individualized decision making and changes decisional balance by providing information about benefits and harms in the context of age and co-morbidities. Using this information to clarify their values, and consider their personal health conditions leads to a change in their beliefs about the benefit of universal screening, and has them consider their personal preferences regarding screening in the context of the benefits and harms. As they consider changing their screening preference, they are activated to discuss their preferences with their provider. Because patients are prepared for individualized decision making, the primary barrier for providers has been addressed. Providers and patients can then engage in individualized decision making which leads to appropriate decision making about CRC screening. Furthermore, because of the individualized decision making process they are more likely to follow their providers' clinical recommendations, resulting in appropriate screening.

Figure 1

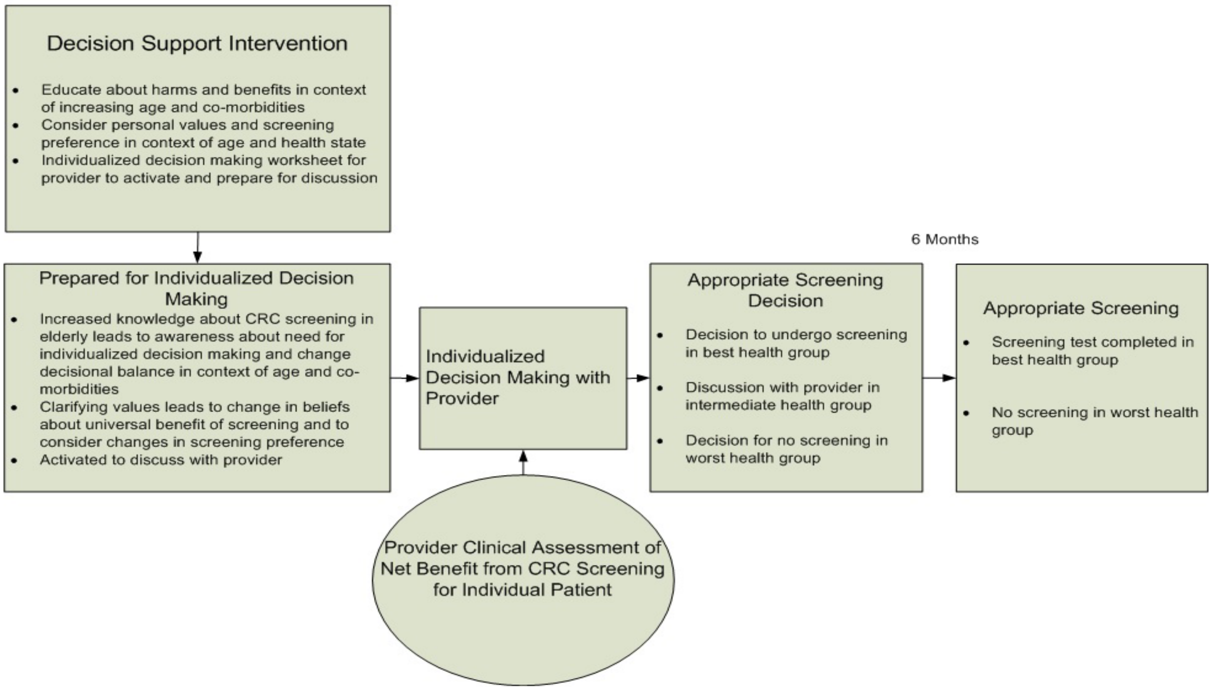

Summary of Significance: The proposed research addresses the safety of an important preventive service, CRC screening in a vulnerable, elderly population. If successful, the approach can be applied to other preventive services in the elderly. The research is responsive to the RFA in the following ways:

- The research will directly address patient safety and the USPSTF recommendations by attempting to target screening in those most likely to benefit and avoiding screening in those least likely to benefit.

- The decision support intervention is designed to translate population based estimates of the potential benefits and harms of CRC screening and target this information to the individual, so that they can participate in individualized decision making with providers given their personal health state and age.
- It will provide new knowledge on how elderly patients perceive the harms and benefits of CRC screening and whether a decision support intervention designed to increase understanding of these tradeoffs can change these perceptions.
- Finally, if successful in changing patient screening behavior to appropriate screening, the results have broad implications beyond CRC screening in terms of resource use and public trust in the health care system.

Innovation

- The proposed research operationalizes a new screening paradigm as proposed by the USPSTF recommendations, shifting from screening test promotion to facilitating appropriate screening.
- The research proposed in this application moves the scientific discourse from simply describing inappropriate screening to exploring ways to improve appropriate screening in the elderly.
- Focusing on appropriate screening is innovative because it addresses both under use of screening in the elderly who are at increased risk of developing CRC cancer, overuse in those who are at risk of net harm, and promotes patient centered care regarding CRC screening.
- The classification of appropriate screening, while carefully derived from the existing descriptive literature, is novel because it can serve as a foundation for future quality improvement research.
- Our conceptual model brings together decision making constructs from psychology and combines them with clinical decision making processes to examine the complexity of clinical decision making.

Approach

Overview: In developing our approach several considerations were paramount. First, we wanted to capitalize on the strengths of our research team in performing RCTs of interventions in clinical practices (Pignone et al., 2000; Lewis, 2010; Pignone et al., 2011; Sheridan et al., 2004; Lewis et al., 2003) and on our preliminary research (Lewis et al., 2008; Lewis et al., 2009; Lewis et al., 2006; Lewis et al., 2010). Second, our measures were designed to be parsimonious to assure success in the primary care setting, yet be clinically relevant and informative. Where possible we used validated measures; however, given that interventions related to this topic are novel, in some cases we had to modify or develop measures used in the preliminary studies. Importantly, to evaluate whether or not we improve appropriate care we developed a classification scheme using the Charlson Comorbidity Index (Charlson et al., 1994) based on emerging descriptive literature defining quality for screening in the elderly (see measures section). Third, we are partnering with the Duke Primary Care Research Consortium (PCRC) to ensure we are able to enroll an adequate sample to answer the research questions (O'Connor et al., 2003; Kroenke et al., 2001; Bosworth et al., 2009; Svetkey et al., 2009; Pollak et al., 2010). Finally, the approach we propose is based on research conducted by our group with both physicians and adults age 70 and older regarding cancer screening in the elderly. Next we will briefly summarize relevant findings for this application.

Figure 2

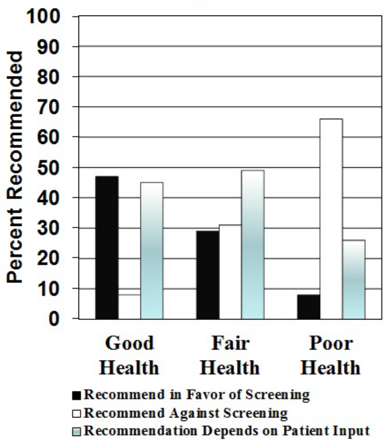

Preliminary Research: Physician Research Results: In two studies we conducted surveying physicians about reported practices in response to clinical vignettes, there were three key findings relevant to this application. 1) Physicians' recommendations clearly varied by health state ($p<0.00$) indicating that physicians endorse individualized decision making 2) a small portion of their recommendations for patients in the good and poor health states were inappropriate, indicating that physicians can perform accurate clinical assessments at each end of the health spectrum 3) a significant portion in each health state would seek patient input (See Figure 2). These results from our national survey (n=276) are pending publication but are similar to our findings at UNC. (Lewis et al., 2008) Our qualitative results interviewing physicians support these quantitative findings (Lewis et al., 2009). Physicians reported using both clinical and individual factors to help in the decision making and emphasized the need to educate patients, so that they could participate in the decision making process.

Patient Research Results: We have completed several descriptive studies of people's perceptions about the

decision to stop cancer screening. As part of the DECISIONS study aimed to assess informed decision making with providers across multiple health conditions (Zikmund-Fisher et al., 2010), we evaluated plans to stop cancer screening among a national sample of 1237 people who recently considered screening with their physicians. Only 12% reported plans to stop cancer screening (12% for breast, 6% for prostate and 9% for CRC). There were no differences between those who were age 70 and older compared to those ages 50 to 70. Importantly, participation in the decision making with providers was strongly associated with plans to stop (Lewis et al., 2010). Our previous in depth interviews with 116 retirement community dwellers revealed similar enthusiasm for continuing cancer screening (Lewis et al., 2006). Participants had inadequate knowledge about harms and benefits of screening with age and comorbidity despite being a very well educated sample. *To address these knowledge deficiencies and perceptions we developed a patient decision support intervention.*

Development of Patient Decision Support Intervention: We based the content of the decision intervention on several conceptual frameworks. The individualized decision making framework proposes that the decision about cancer screening in the elderly depends on an assessment of the potential net benefit from undergoing screening and patient preference (Walter & Covinsky, 2001). Underlying this framework is the more general concept of informed decision making (Braddock et al., 1997; Braddock, 1998). The Ottawa Decision Support Framework was developed by O'Connor and colleagues (O'Connor et al., 1998) to develop tools to assist patients so that they can make informed medical decisions consistent with their personal values. Based on the Ottawa framework and international standards for decision aid development (Elwyn et al., 2006) we developed two components for the intervention (educational and values clarification). From our in-depth interviews with adults age 75 and older (Lewis et al., 2006), we developed 5 key messages for the educational component and nine constructs for the values clarification component. We preformed numerous rounds of cognitive interviewing iteratively updating the intervention. In early rounds we assured that the messages were understandable and not offensive, and in the later rounds we assessed details such as text size, and impressions of layout and graphics (Lewis et al., 2010).

Decision Support Prepared Users for Individualized Decision Making: We conducted a pilot study with pre-post design in community dwellers ages 70 and older demonstrating that the decision support intervention prepared participants for individualized decision making using a combined measure of knowledge and the values clarification subscale of the decisional conflict scale (O'Connor et al., 1998; Mathieu et al., 2007) The rationale for this measure is that elderly individuals must be informed about the potential harms and potential benefits of undergoing screening and have considered their personal values in this decision. We found that the decision support intervention increased the proportion with adequate knowledge (score 10/15 correct) from 4% to 52% ($p < 0.01$) (Figure 3). The proportion prepared to make an individualized decision increased from 4% to 41% ($p < 0.01$). The proportion reporting a preference to undergo CRC screening decreased from 67% to 61% ($p = 0.76$). Seven participants (15%) changed screening preference (5 against screening, 2 in favor of screening) after the decision support intervention. In debriefing, none of the participants reported problems with the content of the material. Importantly, most reported a desire to discuss the decision with their physicians.

Figure 3

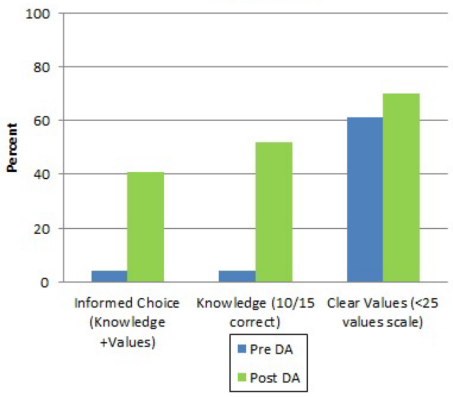

Feasibility Study in Clinical Practice: To test the feasibility of performing an efficacy study in clinical practice, we recruited 5 physicians and consecutive patients of these physicians who were age 70 and older, scheduled for an upcoming appointment with eligibility criteria similar to those described in the recruitment section below. The approach was similar to what we are proposing in this application except that we audio-taped patient physician interactions. We found that audio-taping interactions was a significant barrier to patient recruitment with only 28% of those approached willing to participate, the majority declining because of audio-taping. Prior to decision support, 8 of the 20 patients preferred to undergo screening, 3 preferred no screening and 9 were unsure. Post decision support, 10 patients changed their screening preferences resulting in 6 who preferred screening, 9 preferred no screening, and 5 were unsure. Prior to decision support 10 patients thought the benefits outweighed the risks, 7 thought they were about the same, and 2 thought risks outweighed the benefits. After decision support 13 patients thought risks were greater than the benefits, 2 about the same and

5 thought benefits were greater than the risks. Using the appropriate screening scheme proposed in this application, 12 of 20 of the screening decisions were appropriate according to patient reported outcomes (7 of the 8 inappropriate decisions occurred in the best health state where there was no decision to undergo screening or the decision was deferred). We analyzed audiotapes of the visit to determine visit outcome, specifically whether CRC screening was discussed and if a decision about screening was made. Discussions occurred in 12 of the 20 interactions with 7 discussions in favor of screening (6 of these for FOBT; 1 for colonoscopy), and 5 where the decision was deferred. Patient report of discussions was 100% concordant with the audiotapes; therefore for the proposed study, we will use patient reported outcomes of the discussion.

Practice Recruitment and Physician Education: We will recruit 8 to 10 practices from the Duke PCRC to obtain 40 to 50 providers out of 154 (122 physicians; 32 mid-levels) in the network. This will allow us to recruit up to 10 patients per provider (See sample size calculation). For providers who agree we will hold an educational session to provide information about decision support interventions in general and our decision support intervention in particular. We will discuss USPSTF Guidelines for CRC screening and the individualized decision making framework. The goal of the session is not to improve the accuracy of providers' clinical assessments, as we have found them to be generally accurate. Instead, the goal of these sessions is to prepare them to respond to patients who initiate CRC discussions in response to the intervention.

Eligibility and Recruitment of Patient Participants: For participating providers, the clinical trials coordinators /assistants assigned to participating practices will identify potentially eligible patients using the centralized database consisting of appointment schedules for each provider and the electronic medical record (EMR). They will identify men and women ages 70 to 85 with appointments upcoming within 4-6 weeks and are not up to date with CRC screening, have no previous personal history of CRC, adenomatous polyps, or inflammatory bowel disease according to the EMR. We define not up to date with CRC screening as no evidence of FOBT within 1 year, or sigmoidoscopy within 5 years. We will include those who are not up to date with colonoscopy. These will include patients who have had a previous negative colonoscopy 10 years ago or those who had a negative colonoscopy at least 5 years ago but are due again because on a previous exam they were found to have hyperplastic polyps (Winawer, 2006; Atkin et al., 1992). The rationale for including these patients is that their risk of CRC is similar to those who have not had polyps removed yet are at the same potential risk of harms. Patients who remain eligible after the chart audit will be sent a letter from their providers, explaining the study and allowing them to opt out of further contact. Those who do not opt out will be contacted by phone or approached in the practice to participate. Potential participants will complete a brief eligibility survey. This survey will confirm screening status because the medical records may be incomplete. In addition, the coordinators will confirm that patients speak English, exclude patients with severe sight or hearing impairment that would preclude decision support use and those with possible dementia (Callahan et al., 2002). Those who are eligible and agree to participate will be instructed to come one hour prior to their appointment. On the day of the appointment, participants will be consented, complete the eligibility survey if not done previously on the phone. Those who are eligible will be randomized to intervention or the control arm.

Description of Intervention and Control Conditions: The decision support intervention is a 13 page paper based tool with large font to accommodate visual difficulties with age that was developed and tested by the investigators (Preliminary results). Because the intervention was not as effective in low literacy users in our pilot test, we have revised it for 7th grade reading level (Doak et al., 1996) without changing the content. Because not all provider-patient interactions resulted in a discussion of CRC in our preliminary study, we have added text to motivate patients to discuss the topic with their physicians and added the Individualized Decision Making Worksheet as a paper cue for providers (See Appendix A for example: Targeted to Women Aged 80-85). The decision support intervention takes between 5 and 15 minutes to use. Below is an outline of the content.

1) <u>Educational component</u>: description of FOBT and that all positive stool tests will require a follow-up diagnostic colonoscopy; description of colonoscopy and potential harms; explains the uncertainty of net benefit at the individual level by introducing the concept of competing mortality; explains why individualized decision making is necessary and the need to weigh the harms and benefits of CRC screening; provides a visual demonstration of the balance of benefits and harms by overall health status.

2) <u>Values clarification component</u>: users respond to nine statements defining different constructs that could vary depending on a patient's personal values regarding CRC screening.

3) Individualized decision making worksheet for visit: serves as cue for discussion with providers, indicates patient's personal values through responses on the values clarification component and preference for screening after using the decision support intervention.
Participants will be taken to a quiet research space available in each practice to review materials and complete the surveys. They will complete the booklet and post intervention survey before their visit, and an additional shorter survey after the visit. Six months after the index visit, the coordinators will contact the participants by phone for the final survey. Participants will receive $50 for participating ($25 after visit, $25 after phone call). The procedures will be identical for the control group patients except they will be provided a written booklet on medication management as an attention control instead of the CRC decision support intervention.

Measures, Data Collection, and Data Analysis

Table 5 summarizes the measures for the study. The sources of the data are patient reported.

**Table 5** **Measures**	**Eligibility Screening/ Baseline Measures**	**Post Intervention Survey**	**Post Visit Survey**	**Phone Survey at 6 months**	**Source of Measure**
**Eligibility measures**					
Dementia Screen	X				Callahan et al., 2002
Not up to date with CRC screening	X				Pignone et al., 2000
**Measures to Classify Patients into Appropriate Screening Groups**					
Co-morbidity Index	X				Katz et al., 1996
Age group	X				
**Outcomes**					
Main Outcome Aim #2<br>Appropriate CRC screening 6 months after index visit			CRC discussion during index visit	Screening test completed and type of test completion vs. no test completion	Walter, 2009; Kistler, 2011; Kahi, 2009; Charlson et al., 1994
Secondary outcome Aim #1: Appropriate screening decisions after index visit			CRC Discussions and screening decisions		Walter, 2009; Kistler, 2011; Kahi, 2009; Charlson et al., 1994
**Decision Making Outcomes/Mediating Variables**					
Prepared for Individualized Decision making (Knowledge score+ Values Clarification subscale)		X			Lewis et al., 2010; O'Connor et al., 1998
Screening preference		X			Preliminary Study
Balance of harms and benefits (decisional balance)		X			Preliminary Study
Intent to discuss CRC screening during the visit		X			Pignone et al., 2000
**Covariates**					
Demographics: Age, race/ethnicity, gender, education/SF 1	X				
Previous CRC screening (ever)	X				
Literacy level		X			"REALM-SF", n.d.
Visit type		X			
Saw regular physician			X		

- See Appendix C for examples of surveys

Aim 1) To examine the effect of a patient decision support intervention on appropriate screening decisions in patients ages 70 to 85 immediately after the index visit with their primary care providers: Appropriate screening decisions will be determined from patient reported information immediately after the visit. For this study we have developed a classification scheme to assess appropriate screening decision making. It is a combined measure using patient reports about the screening decision outcome and discussions about CRC screening immediately after the index visit. The appropriate outcome depends on the patient's age and co-morbidity level according to the Charlson Comorbidity Index (Charlson et al., 1994). The Charlson Comorbidity Index (CCI) is the most commonly used long-term measure of comorbidity and mortality (de Groot et al., 2003). An increase of 1 in CCI score approximately increases the risk of death to that seen from an additional decade

of age (Charlson et al., 1994). While other ways of measuring comorbidity and mortality exist, the CCI has been found to be more feasible and reliable than other measures in regards to mortality (de Groot et al., 2003; Shack et al., 2010; Hall, 2006). Classification for appropriate screening by age and comorbidity level is given in Table 6. The classification scheme is based on the life expectancy needed to expect a net benefit from screening (Walter & Covinsky, 2001; Gross et al., 2006). RCTs of CRC screening suggest that patients must be expected to live at least 5 years to have the potential to benefit from screening (Hardcastle et al., 1996; Kronberg et al., 2004; Mandel et al., 1993), though this number may well be closer to 10 years (Winawer et al., 1992; Winawer et al., 1991; Kozuka 1975; Chen et al., 2003). Using this as a basis, we reviewed the descriptive literature that has emerged defining quality standards (Walter & Covinsky, 2001; Braithwaite et al., 2009; Gross et al., 2006; Kahi, 2009; Kistler et al., 2011; Schneeweiss et al., 2003; Fisher et al., 2007). From this literature, we chose longitudinal cohort studies that categorize patients by age and co-morbidity from the CCI because they could be used easily in a clinical setting (as opposed to predictive modeling) (Walter, 2009; Kistler et al., 2011; Kahi, 2009). Although the USPSTF recommends that individualized decisions start after age 75, these studies show limited life-expectancy in the sickest patient ages 70-74. Using the individualized decision making framework (Walter & Covinsky, 2001; Fraenkel & Fried, 2010) we defined the best health group as those who are youngest with the least comorbidities where all three studies agreed that screening was likely beneficial, the worst health group who are oldest with the worst Charlson scores where all three studies agreed that screening was likely not beneficial, and the intermediate group where there was disagreement or the studies could not say whether or not screening would be of net benefit or harm. The CCI score will be derived from patient self-report which is a well-validated analog to the original chart-based Charlson Comorbidity Index (Katz et al., 1996).

**Table 6**		Charlson Comorbidity Score		
		0	1-3	≥4
Age	70-74	Screen	Screen	Discuss
	75-79	Screen	Discuss	No Screen
	80-84	Discuss	No Screen	No Screen

Aim 2) To test the effect of a patient decision support intervention on appropriate CRC screening six months after the index visit with their providers. We will determine screening test completion or no screening test completion by patient report six months after the index visit. We anticipate that test completion could differ from the decision making outcome from aim #1 because patients in different health groups could differ in screening uptake. Our approach to assess appropriate screening for aim #2 will be identical to the secondary outcome in aim #1 except the appropriate measure will use patient reported screening test completion or non-completion and patient reported discussions about CRC screening previously measured after the index visit.

Aim 3) To examine the extent to which patient decision making factors mediate the impact of the intervention on appropriate CRC decisions and screening: A key aim of the proposed study and of the RFA is to understand how patients evaluate approaches to improve risk communication and assess patients' perceptions of harms. To address these objectives, this aim will to assess mediators of the patient decision making process. In our preliminary studies we have piloted these measures and have shown that they change after participants have used the decision support intervention.

Statistical Analyses: The statistical analyses will be performed as intention to treat basis. For aim 1, we will first examine the distribution of ages by Charlson comorbidity score separately by study arm. We will compare the arms at baseline with respect to other potential confounders. We will then calculate the secondary outcome of the proportion of appropriate screening decisions per arm, and will use a mixed effects logistic regression model with intervention arm as the only covariate and a random intercept for physician within practice to test the effectiveness of the intervention while accounting for the correlation among participants within a physician/practice. Further models will incorporate other covariates that may account for residual confounding of the intervention effect. For Aim 2; the statistical regression analysis methods will be similar to aim #1 except for these mixed effects logistic regression models the primary outcome of appropriate screening will be used. Once we have tested our hypothesis for the main effects, we will also perform hypothesis-generating exploratory analyses to examine effects in sub-groups of patients. We will examine the effect in the three strata (best, intermediate, worst health groups) defined by age and Charlson comorbidity score. We will also examine other sub-groups including gender, previous screening, and literacy level. We also plan an exploratory analysis examining type of screening test completed. In our preliminary results we found that FOBT was chosen 6 out of 7 patients who decided in favor of screening. Typically FOBT is chosen only 12% of the time at the UNC Internal Medicine practice, suggesting a move to less aggressive screening. For Aim 3, analyses will once again use mixed effects logistic regression modeling, but now will consider the covariates mentioned above as potential mediators using approaches described by MacKinnon et al. (MacKinnon et al., 2002). The potential mediators to be examined include adequate preparation for individualized decision making (adequate

knowledge+ clear values); knowledge scores, screening preference before the index visit, decisional balance, and reported discussion during the index visit.

Sample size considerations and randomization: For this hierarchical structure 2-arm intervention study, we must account for the correlation among patients within a physician/practice. From prior work, we conservatively estimate this intraclass correlation coefficient (ICC) to be 0.0225. The smallest meaningful increase in percent of "appropriate screening" is judged to be 15%. Given that we anticipate that recruitment expectations place a cap of patients that can be recruited in a period of 24 months to roughly 400-450 patients total, we calculated a range of possible scenarios with the following assumptions: 1) n = 400 or 450 total patients (=200 or 225 patients per arm since using a 1:1 allocation ratio) 2) ICC = .01, .02, or .03 3) Meaningful effect =.15 or .20 4) Proportion appropriately screened in Control arm = 0.50 (most conservative) With m=10 or 15 patients per doctor, the VIF = 1 + (m-1) ICC ranges and the "effective sample sizes" are shown in the tables with (a) Likely intraclass correlation coefficients and (b) effective total sample sizes. So the 'largest effective total sample size is 413 and the smallest is 282 (=206 to 141 per arm). The power that we can obtain for the meaningful effect sizes of 20% and 15% are given in the plot. (N Query 6.0): Thus, if our intervention increases appropriate screening by 15% (solid curve), with 180 patients per arm we will have 80% power, while if our intervention is more effective (20% increase), we would only need 100 patients per arm. With an expected ICC of 0.02, our effective sample size is 169 per arm, with power about 80% if our increase is only 15% but over 90% if it is closer to 20%. Among 10 practices from the Duke PCRC we anticipate 15,000 age eligible patients. Conservatively assuming that only 25% meet our eligibility criteria and only 50% agree to participate we should have 1875 patients available over the two year period. We thus plan to recruit 450 patients from 45 physicians (10 patients per physician). The PCRC has an excellent record retention record (88%) (Svetkey et al., 2009; Bosworth et al., 2009) so we anticipate retaining at least 400 of the 450 patients at 6 months. Randomization will be done centrally at UNC. Because of our interest in studying the 'worst health' group, randomization will be stratified by health status into best, intermediate and worst health groups to ensure adequate numbers in each subgroup. Within each strata, permuted blocks will be used to assign participants to intervention or control arm.

ICC → m ↓	.010	.020	.030
10	1.090	1.180	1.270
15	1.140	1.280	1.420

n → VIF	400	450
1.090	367	413
1.140	351	395
1.180	339	381
1.270	315	354
1.280	313	352
1.420	282	317

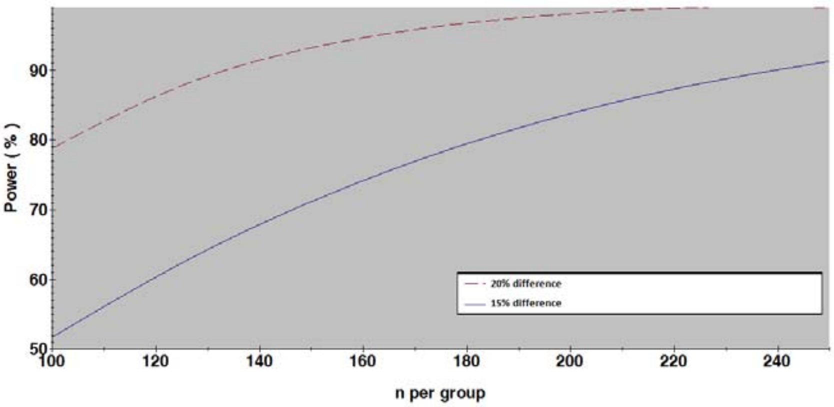

Training, quality control, and coordination among the sites and data management

Dr Lewis and co-investigators will modify the previous study protocol from the preliminary study for use by the Duke PCRC staff. Usual procedures per the PCRC will be implemented for staff training and quality assurance. The PCRC will manage the recruitment and tracking database as these data include patient health information and will not be shared across sites. De-identified data will be transferred to UNC for analysis.

Timeline: For a timeline of project 3, please see Table 3 on page 127.

Limitations and contingencies: We considered using chart reviews in addition to patient self-report to determine screening outcomes but budgetary considerations would not allow us to do so. After careful consideration, we chose patient self-report even though it has some limitations because we were more concerned about bias introduced by incomplete medical records, especially for records that demonstrate no screening. We anticipate having an adequate pool of participants; however, if we are not reaching our target goals within three months of starting recruitment we will perform additional rounds of provider recruitment within our existing practices as not to increase costs. Provider contamination is a theoretical concern; however given that educational interventions have not succeeded in changing provider behavior we doubt that this will be an issue. We have proposed reasonable classifications for appropriate screening using the existing descriptive literature defining quality. Using these classifications and a randomized design, we are able to test the efficacy of the patient decision support intervention proposed in this application with good internal validity.

However, these proposed cut points are not meant to as a permanent standard from which quality of care is to be defined, but rather as a benchmark from which to launch new research aimed to improve appropriate screening.

Summary: The proposed research will directly address patient safety and the USPSTF recommendations by attempting to target screening in those most likely to benefit and avoiding screening in those least likely to benefit. In doing so, the research proposed in this application moves the scientific discourse from simply describing inappropriate screening to exploring ways to improve appropriate screening in the elderly. If successful in changing patient screening behavior to appropriate screening, the results have broad implications beyond CRC screening in terms of resource use and public trust in the health care system.

Summary

Pieces of a Puzzle

To reduce the potential harms of C, D, and I clinical preventive services, we must develop innovative research methods and concepts; we must train investigators in these methods; and we must conduct the research needed to better understand how patients and physicians think about harms and how they use (or do not use) information about potential harms in decision making. Then we must design and test strategies for increasing appropriate decisions in controlled and then wider settings. Finally, we must disseminate effective strategies and educate physicians in training about appropriate decision making. The research steps to reducing the potential harms are shown in Table 7. Our research program is a strong beginning that moves us forward on this agenda.

Table 7: Research Steps to Reducing Potential Harms of C, D, and I Clinical Preventive Services*

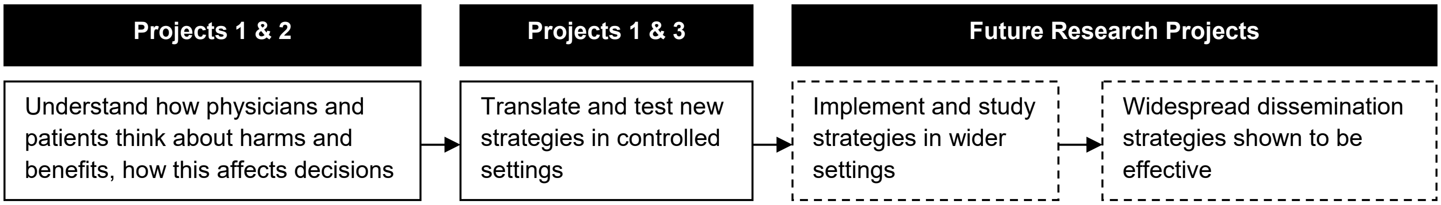

1. Need to understand (Project 1: patients; Project 2: physicians and patients)
   a. Patient/clinician knowledge of/thinking about harms of clinical preventive services
   b. How patients/clinicians consider harms of potentially harmful clinical preventive services in making decisions about use; whether such consideration involves weighing benefits and harms
   c. Whether and how patients/clinicians view harms in relation to benefits in making decisions
   d. What factors beyond balance sheet benefits and harms influence patient/clinician decisions
   e. How patients and clinicians finally make decisions about use of clinical preventive services
2. Need to understand (Projects 1 and 3: patients)
   a. Effectiveness of decision support of various "types" (i.e., mode of delivery, time of delivery, use of written vs. video, etc) in increasing appropriate decisions
   b. Effectiveness of various types of content (i.e., key messages, ordering of information and messages, skills building) of decision support in increasing appropriate decisions
   c. Effectiveness of different "targets" (i.e., patient, clinician, both) of decision support
3. Translate understanding into new strategies; test in controlled settings (Project 3)
4. Implement and study likely effective strategies in wider settings
5. Widespread dissemination of strategies shown to be effective in increasing appropriate decisions

*: All of these may vary with the specific clinical preventive service being considered. Our projects together will help us understand the extent to which effective strategies vary among different services.

In addition to this research agenda, our Core Office will move the educational and dissemination agenda forward so that the understanding that we now have and that we gain from research can be translated into action at the practice and policy levels. Our ReCPS proposal will lead a major effort in research, in dissemination, and in education to broadly increase the visibility of this issue, and to finally reduce the overuse of C, D, or I services. We have assembled an outstanding team within an organized structure to lead a exceptional effort to reduce the potential harms of clinical preventive services.
