# Supplementary material for: Effect of a Patient Decision Aid on Preferences for Colorectal Cancer Screening Among Older Adults: A Secondary Analysis of a Randomized Clinical Trial
Source: JAMA Netw Open. 2022 Dec 5;5(12):e2244982. doi: 10.1001/jamanetworkopen.2022.44982 (PMC9855297; doi:10.1001/jamanetworkopen.2022.44982)
Supplement: Supplement 2. — Trial Protocol [file jamanetwopen-e2244982-s002.pdf]

EPIC-D

Elders' Preferences in Care Decisions

University of North Carolina at Chapel Hill
And
Duke University

STUDY MANUAL AND PROCEDURES

Version 4.0

May 22, 2013

Manual Content:

Principal Investigator: Carmen Lewis, MD, MPH

Co-Principal Investigator at Duke: Rowena Dolor, MD, MHS

Lisa Werner, MA Ed.

University of North Carolina at Chapel Hill

School of Medicine

Division of Internal Medicine

Chapel Hill, NC 27599

Tel: 919-966-1584

Fax: 919-966-1634

Duke Primary Care Research Consortium

Division of Internal Medicine

Durham, NC

TABLE OF CONTENTS

Study Summary …… 1

GOAL…… 1

SITE …… 1

RANDOMIZED CONTROLLED TRIAL …… 1

POPULATION…… 1

SAMPLE SIZE…… 2

DURATION…… 2

PRIMARY OBJECTIVES…… 2

DESCRIPTION OF INTERVENTION AND CONTROL CONDITIONS …… 3

Methods …… 3

PURPOSE…… 3

APPLICABILITY…… 3

RESPONSIBILITY…… 4

A. Principal investigator…… 4

B. Co-Investigator…… 4

C. UNC project manager…… 4

D. Duke project lead…… 4

E. Duke project coordinator…… 4

F. Duke study staff…… 5

PROCEDURES PRIOR TO PATIENT APPOINTMENT …… 5

A. Obtaining information from electronic medical record……5

B. Process for finding eligible patients…… 5

C. Chart reviews……6

D. Eligible patients after chart review and in the future…… 7

E. Mailings and Phone Calls …… 8

Mailing invitation letters and recruitment mail ……8

F. Screening for eligibility by Phone/Study Recruitment …… 8

Phone calls……8

G. Pre-screening by Phone…… 9

Eligibility survey……9

Re-contacting patients……11

Baseline survey……11

DAY OF PATIENT APPOINTMENT 12
A. Informed Consent........ 12
B. Patient Randomization 13
Health states........ 13
C. Assigned Randomization Number........ 13
Randomization of education tool........ 13
D. Post Intervention Survey........ 14
E. Post Visit Survey........ 14
AFTER THE PATIENT VISIT 15
A. Storage of Study Forms........ 15
B. Six Month Follow Up........ 15
C. Subject Reimbursement........ 15
Data Management 16
CHART ABSTRACTIONS 17
QUALITY CONTROL........ 17
Appendix 1: Summary of Study Events 18
Appendix 2: Study Organizational Chart 20
Appendix 3: List of All Study Forms........ 21

List of acronyms

CRC	Colorectal Cancer
DCRI	Duke Clinical Research Institute
EMR	Electronic Medical Record
EPIC-D	Elders’ Preferences in Care Decisions
ETR	Employee Travel and Reimbursement
FOBT	Fecal Occult Blood Test
IBD	Inflammatory Bowel Disease
ICF	Informed Consent Form
PCRC	Primary Care Research Consortium
PI	Principal Investigator
PM	Project Manager
RA	Research Assistant
SOP	Standard Operating Procedure
QC	Quality Control
UNC	University of North Carolina at Chapel Hill
URN	Unique Research Number
USPSTF	United States Preventive Services Task Force

Study summary

Goal

Study researchers propose a randomized controlled trial at the patient level to determine the efficacy of the intervention within a clinical setting. We hypothesize that the use of the intervention will prepare patients for individualized decision making with their providers and result in an improvement in appropriate CRC (Colorectal Cancer) screening decisions and screening outcomes. To assess appropriate CRC screening decisions and screening, we will use a classification scheme derived from the literature based on age and the Charlson Comorbidity Index. Using this scheme, appropriate screening will include screening for those in the best health because they are likely to benefit, no screening for those in the worst health because they are unlikely to benefit, and evidence of a discussion about CRC screening for those in the intermediate health group because the benefit is unclear.

The proposed research will directly address patient safety and the USPSTF recommendations by attempting to target screening in those most likely to benefit and avoiding screening in those least likely to benefit. It will provide new knowledge on how elderly patients perceive the harms and benefits of CRC screening and whether a decision support intervention designed to increase understanding of these tradeoffs can change these perceptions. Finally, if successful in changing patient screening behavior to appropriate screening, the results have broad implications beyond CRC screening in terms of resource use and public trust in the health care system.

Site

The trial will be conducted at Duke Primary Care clinics through the Duke Primary Care Research Consortium: a large North Carolina network consortium based in Durham, NC. Participants will be asked to read the health information targeted for older adults and complete a few brief questionnaires. Participants are randomly assigned to one of 2 groups, an intervention or an attention control.

Randomized Controlled Trial

This study will involve participants from primary care settings who are part of the Duke Practice Based Research Network. We will recruit approximately 45 physicians within the network and about 10 patients from each physician for a total of 450 patients.

Population

English speaking men and women ages 70 to 84.

- No previous personal history of CRC
- No inflammatory bowel disease
- Not up to date with colon cancer screening or surveillance

Included are those patients who are not up to date with colonoscopy. These will include patients who have never had a colonoscopy, had their last colonoscopy at least 10 years ago, who have not had a colonoscopy within the last 4 years, and those who had their last colonoscopy between 4 and 10 years ago but are due now for their next colonoscopy.

Sample Size n = 450

Duration

The grant period for the entire grant including recruitment is September 29, 2011 through September 29, 2014. Recruitment will last until approximately December 2013.

Primary Objectives

Hypothesis for all the Aims

Aim 1) To examine, in a randomized trial, the effect of a patient decision support intervention on appropriate screening decisions in patients ages 70 to 84 immediately after the index visit with their primary care providers.

The intervention group will have a higher proportion of patients in which the decision about CRC screening is classified as appropriate. Specifically, the proportion of intervention patients in the worst health group who plan to be screened will be lower while the proportion of intervention patients in the best health group who plan to be screened will be higher than analogous patients in the control group. For the intermediate health group, intervention patients will report more discussions regarding CRC screening than control patients after the index visit.

Aim 2) To test the effect of a patient decision support intervention on appropriate CRC screening six months after the index visit with their providers. Because screening uptake at six months may differ significantly from screening decision outcomes, we will assess screening test completion. Patients in the intervention arm will report a higher proportion of CRC screening classified as appropriate. Specifically, intervention patients in the worst health group will have lower screening rates while intervention patients in the best health group will have higher screening rates than analogous patients in the control group. For the intermediate health group, intervention patients will report more discussions regarding CRC screening than control patients after the index visit.

Aim 3) To examine the extent to which patient decision making factors mediate the impact of the intervention on appropriate CRC decisions and screening. We hypothesize that the intervention will have more effect when patients are prepared for individualized decision making (demonstrate adequate knowledge and clear values) and when patients' decisional balance and screening preferences align with their particular classification for appropriate screening given their age and

health state. We also hypothesize that discussions about CRC screening with providers are necessary for the intervention to be efficacious.

Description of Intervention and Control Conditions

The decision support intervention is a 13 page paper based tool with large font to accommodate visual difficulties with age and is at the 7th grade reading level. Also, we have added text to motivate patients to discuss the topic with their physicians and added the CRC Supplement as a paper cue for providers (See Appendix 3 for Randomized Arm-Educational Materials). The decision support intervention takes between 5 and 15 minutes to use. Below is an outline of the content.

1) Educational component: description of FOBT and that all positive stool tests will require a follow-up diagnostic colonoscopy; description of colonoscopy and potential harms; explains the uncertainty of net benefit at the individual level by introducing the concept of competing mortality; explains why individualized decision making is necessary and the need to weigh the harms and benefits of CRC screening; provides a visual demonstration of the balance of benefits and harms by overall health status.

2) Values clarification component: users respond to nine statements defining different constructs that could vary depending on a patient's personal values regarding CRC screening.

3) Individualized decision making worksheet for visit: serves as cue for discussion with providers, indicates patient's personal values through responses on the values clarification component and preference for screening after using the decision support intervention.

Participants will be taken to a quiet research space available in each practice to review materials and complete the surveys. They will review the booklet and complete the supplement before their visit.

The procedures will be identical for the control group patients *except* they will be provided a written booklet on older adult driving as an attention control instead of the CRC decision support intervention.

Methods

1 Purpose

This Standard Operating Procedure (SOP) applies to procedures related to the EPIC-D study as carried out by the study team members at Duke PCRC.

2 Applicability

All study team members involved in the recruiting and follow up of subjects for EPIC-D should comply with these procedures.

3 Responsibility

3.1 **The Principal Investigator (PI),** Carmen Lewis (Carmen_Lewis@med.unc.edu) is located at The University of North Carolina at Chapel Hill. As the principal investigator, she will be responsible for all aspects of the project. Specifically, she will oversee the data collection, data management and analyses. Working with Dr. Dolor and the Duke PCRC staff, she will be responsible for implementing all aspects the research protocol.

3.2 **The Co-Investigator**, Rowena Dolor, (Rowena.Dolor@duke.edu) is located at Duke Primary Care Research Consortium (PCRC). She is the Director of the PCRC. Dr. Dolor will assist with practice site recruitment and interact with the physicians at the practice sites to assure quality, appropriate study monitoring and adherence to the study protocol. She will ensure that the protocol is implemented appropriately by Duke staff at the practice sites.

Dr. Dolor will be responsible for overseeing patient recruitment at Duke to reach study targeted recruitment goals.

3.3 **The UNC Project Manager**, Lisa Werner (Lisa_Werner@med.unc.edu) will ensure that UNC IRB, study SOP, study documents and reports are submitted to appropriate agencies. Dr. Lewis and the project manager will conduct bimonthly project meetings via phone conference. The purposes of these calls will be to ensure that the protocol is implemented appropriately, to update on recruitment and data collection progress, and to review and disseminate results.

Ms. Werner will assist in training Duke staff for study start up on the study protocol. She will also be the UNC study super administrator for the database system. She is also responsible for maintaining the interface with the research team for administrative issues and project timelines.

3.4 **Duke Project Lead**, Virginia Beth Patterson, is a Project Leader for the PCRC (Virginia.Patterson@duke.edu). Ms. Patterson will serve as the project leader for Duke and be responsible for overseeing the management and coordination of PCRC site activities including protocol education, protocol compliance, patient enrollment and data collection. She will also help to assure the monitoring and quality of the study in adherence to the study protocol by submitting weekly reports to UNC. In addition, she will work closely with Dr. Lewis and Dr. Dolor to meet trial timelines and ensure quality deliverables at Duke.

3.5 **Duke Project Coordinator**, Lynn Harrington is a nurse and Clinical Research Coordinator III in the Primary Care Research Consortium (Lynn.Harrington@dm.duke.edu). As a CRC III, Ms. Harrington will be the primary coordinator responsible for patient recruitment and follow-up as well as data collection. She will coordinate the PCRC staff to maximize recruitment

in the most efficient manner. Ms. Harrington will be responsible for training new study staff after study start up. She will work closely with Dr. Lewis and Dr. Dolor to meet trial timelines and ensure quality deliverables.

Ms. Harrington will assist Dr. Dolor in the responsibility of assuring that the protocol is followed at the clinic sites and that study staff are meeting projected recruitment goals and data collection is done appropriately. She will also help to assure the monitoring and quality of the study in adherence to the study protocol by submitting weekly reports to UNC. Source documents for the study containing PHI will be stored and locked in the Duke research staff office.

3.6 **Duke study staff** assigned to the study will use DEDUCE and DISCERN to look for eligible study patients. Staff will enter chart reviews into the database as well as patient information and the patient eligibility survey. Duke study staff will also be responsible for calling participants for study eligibility and recruitment. They will update the tracking database daily to reflect chart reviews, mailing of recruitment letters, phone calls to participants for recruitment, appointment reminders, and 6 month follow up.

4 Procedures Prior to Patient Appointment

4.1 Obtaining Information from Electronic Medical Records

The informed consent form is written according to the Duke IRB template guidelines and approved by the Office of Human Research Ethics (OHRE). A HIPAA Authorization Form is also created in order to allow the Duke research team to get information from medical records to prescreen in the current research study.

4.1.2 Process for Finding Eligible Patients

To search for potentially eligible patients, the Duke staff uses a program called DEDUCE (Duke Enterprise Data Unified Content Explorer). The program allows researchers to query data compiled into the Decision Support Repository (DSR). Anytime a patient is seen at a Duke clinic or hospital, information about the patient and the appointment is added to the DSR. Researchers can then use DEDUCE to filter through all patients seen at Duke and creates a list of those meeting their specified criteria. The database also offers a program called DISCERN (Duke Integrated Subject Cohort and Enrollment Research Network), which can be used to search for future appointments of patients identified through DEDUCE.

Within DEDUCE, Duke staff first has the database create a cohort of all patients previously seen at Duke. From this group, staff selects those patients born between 1927 and 1942 to identify those patients as potentially age-eligible or EPIC-D. Duke staff has the database create cohorts of age-eligible patients previously seen at the particular clinics used for recruitment of the EPIC-D study. With each of these groups, staff create lists of future appointments for the patients identified by using the DISCERN function. For each clinic, study staff

will use DISCERN to find out which patients previously seen there have appointments scheduled at any Duke clinic within the next 28 days. Before a schedule pops up, DISCERN has staff select which clinic to find future appointments for, as well as which specific providers to show appointments for. Staff should select the particular clinic and provider names from a drop-down list, and then DISCERN will provide a schedule. Once a provider has reached the saturation point with 12 patients, then staff won't choose his/her name from the drop-down list.

Once these lists are printed, study staff excludes any patients who were born in 1927 who have already turned 85, as well as any patients born in 1942 who will not have turned 70 by the date of their next appointment. Duke staff then excludes any patients with appointments before 9 a.m., any patients who are coming in for lab-only visits, and any patients whose appointments are solely for Coumadin checks. From the remaining list, study staff checks the medical record numbers (MRNs) of the patients in the EPIC-D database to identify those who have not been previously screened.

4.1.3 **Chart Reviews**

The Duke study staff will identify potentially eligible patients using DEDUCE and DISCERN to find potentially eligible patients, and then check their eligibility with e-browser, the online medical record system and IDX, Duke's s appointment scheduling system.

Study staff will use a chart review (See Appendix for chart review) form to help them with identifying potential study patients. The purpose of the chart review is to determine if a patient is eligible for additional screening for the study. Study staff will look in the patient's EMR for the following information and record answers in the study database. If the patient is found eligible based upon the chart review, a paper chart review is completed and stored in a locked room at the Duke PCRC office. (See Appendix for chart Review Procedures for Duke EMR system).

1. Date of birth- Age eligibility-patient must be age 70 to 84. The programming in the database has been set to the patient's age at 8 weeks after chart review. The exception is for those patients that will age out (turn age 85) before their visit. Patients need to be excluded if they will turn 85 within 8 weeks of the chart review date
2. English as primary language
3. Cognitive eligibility- Review chart for Mini-Mental State Exam (MMSE). If they have a score less than 24 they are ineligible
4. Colorectal Cancer Screening Eligibility and diagnosis of colon cancer. If they have had colon cancer they are *ineligible*
5. Inflammatory bowel disease including Crohn's disease or ulcerative colitis. If they have either type of inflammatory bowel disease they are ineligible.
6. NOT up to date for colon cancer screening or surveillance tests.

Colonoscopy

- o If most recent colonoscopy was 10 years ago or more, they are eligible for the study
- o If the most recent colonoscopy was less than 4 years ago, they are NOT eligible for the study
- o If the most recent colonoscopy was done 4 or more years ago and less than 10 years ago, then the results of the study will determine if they are eligible. Review the results of the colonoscopy to determine the follow up interval

Flexible sigmoidoscopy/Sigmoidoscopy or CT Colonography

- o If they have completed a flexible sigmoidoscopy or CT Colonography in the last 5 years, they are ineligible.

To determine screening dates for colonoscopy, sigmoidoscopy, FOBT, CT Colonography:

- o If only a year is given in the patient's EMR, then enter the default month as January and the day as the 1st of the month.
- o If a month and year are given in the patient's EMR, then enter the default day as the 1st of the month.

Patients not eligible for the study after the chart review will have no paper chart review completed. All eligible and ineligible patients are entered in the database. However, ineligible patient's protected health information will become de-identified in the database. Also, study staff will code the patients as ineligible for the study in the database.

For Duke patients that may become eligible in the future based on their chart review, their medical record number will remain in the database. If the patient appears on a schedule at a later date, study staff will enter the patient's MRN from the providers list of scheduled patients into the database. The patient is eligible or ineligible for the study based on an updated chart review.

4.1.4 **Eligible Patients after Chart Review and in the Future**

Patients that are eligible after the chart review will have their information entered into the study database for prescreened eligible patients. (See Appendix for data entry procedures).

Some patients will be ineligible at their initial chart review but may become eligible in the future. Each time a Duke staff print DISCERN schedules, they will check the MRNs in the database to determine if the patients have been screened before. Patients who have been screened before but who are due for a re-screening, meaning their last appointment was at least 3 months ago, will pop up in the database as "eligible for re-screening." If the patient is eligible for re-

screening, the chart tab will auto-populate with the original chart data so the RAs can edit as necessary for patient eligibility.

If the patient is eligible after initial chart review or a later chart review, at three months, the prescreened patient will be mailed a study invitation letter and study staff will follow up with the patient on the phone for possible study recruitment.

4.1.5 **Mailings and Phone Calls**

Mailing Invitation Letters and Returned Mail

An alert flashes on the chart review page to let Duke staff know how many patients each provider has recruited. If the provider has 12 patients enrolled, Duke staff does not continue with the chart review to ensure the patient's provider is not full and is still accepting eligible patients for the study. If the Duke provider is not full, then patients who remain eligible after the chart review will be sent a study invitation letter from their provider, explaining the study and instructing them to call Duke staff over the phone if they would like to opt out of further contact from the study. The letters will be printed and sent on a daily basis and will be sent out 2-4 weeks prior to scheduled practice site appointments.

Mailed study letters that are returned to sender and undeliverable by the postal service will have their status marked in the database on the patient information tab as undeliverable by study staff. The database will be updated appropriately by study staff to reflect undeliverable status. All other protected health information about the patient will be de-identified in the database.

Study staff should have the following materials in front of them before any mailings are conducted: 1) white envelopes 2) access to the EPIC-D database and a printer and 3) a roll of stamps. Study staff will print invitation letters from the EPIC-D database using mail merge. (See Appendix for data entry procedures).

Study staff will print the study invitation letters and labels from the EPIC-D database using mail merge. Staff will place the study invitation letter into a white envelope with the appropriate patient address and place a stamp on the envelope and mail.

4.1.6 **Screening for Eligibility by Phone/Study Recruitment**

Phone Calls

Duke staff should have the following materials in front of them before any calls are placed: 1) phone script 2) chart review 3) eligibility survey 4) baseline survey and 5) a computer with access to Duke EMR and the EPIC-D database.

Using the patient information in the EPIC-D database, study staff can see the contact information for the patient and document the outcome of the call under the contact section of the database. Study staff should also document in this

section under contact method, “participant” when a patient calls them to opt out of the study or for other information.

Patients that don’t opt out of further contact from study staff from the mailed invitation letter will be called on the phone a minimum of 3 times before their scheduled provider’s visit and assessed for study interest and eligibility. Those patients who are reached will be given more information about the study and asked whether or not they are interested in participating in the study.

Patients interested in participating in the study will be given the eligibility survey over the phone by study staff. (See next section). Patients who opt out over the phone will be thanked for their time and will no longer be contacted. Their information will be updated in the database reflecting opt out status. (See Appendix data entry procedures for instructions).

4.1.7 **Pre-screening by Phone**

Eligibility Survey

Those patients who do not opt out of the study from the recruitment mailing letter or re-contact letter will be contacted by phone by study staff approximately 10 days after the letter are mailed. Staff will explain that the purpose of the study is to test health information made especially for older adults. We want to see if this information is easy to understand and useful.

The first step is to determine whether patients are eligible for the study. Participants must speak English as their primary language, be age 70 to 84, be overdue for screening or due for screening now and not have dementia. (To determine if a patient has dementia, refer to the chart review for the Mini-Mental State Exam (MMSE). If the patient’s EMR showed a score less than 24 for the MMSE, the patient is ineligible.)

Patients must NOT have had colon cancer or inflammatory bowel disease. Inflammatory bowel disease includes a diagnosis of Crohn’s Disease or ulcerative colitis. The reason we want to exclude these groups is because they need a colonoscopy more frequently than the people we are trying to recruit into our study. (See chart review and Eligibility Survey).

Study staff needs to determine if participants are due for colon cancer screening or surveillance colonoscopy. (Refer to the eligibility survey in Appendix). Patient’s that are being re-contacted should have some of the eligibility survey data in the database from prior phone eligibility contact. To determine screening dates for colonoscopy, sigmoidoscopy, FOBT, CT Colonography:

- If only a year is given in the patient’s EMR, then enter the default month as January and the day as the 1st of the month.
- If a month and year are given in the patient’s EMR, then enter the default day as the 1st of the month.

Duke study staff must be very familiar with the flow and the questions on the eligibility survey because some of the questions may be difficult for some people to remember. It is important for study staff to use multiple methods to prompt the participants' memory for the screening dates. (See probes in the Eligibility survey in the Appendix).

In the comorbidity questionnaire section of the survey, some people may ask what certain conditions or diseases are while study staff is going over the eligibility survey. In general, people who have these conditions or diseases will almost always know it. So if the patients don't know the names of these conditions or diseases, there is a very high likelihood that they don't have the disease(s) or condition. (See the Comorbidity Questionnaire section of the Eligibility survey in the Appendix).

Patient eligibility is determined by the results in Sections I, II, and III of the eligibility survey. (English Language Eligibility Passed, Age Eligibility Screener Passed, and CRC Screening Eligibility Passed.) If patients fail ONE of these screeners then they are ineligible for the study.

To determine if the patient's eligibility by health group is either best, intermediate or worst, use a combination of the total comorbidity score from the comorbidity questionnaire and the patient's age from Section I of the survey.

	Co-morbidities Score		
	0	1-3	≥4
70-74	Best	Best	Intermediate
75-79	Best	Intermediate	Worst
80-84	Intermediate	Worst	Worst

Study staff will need to double check with the Health Group Status weekly report from the database (in process) to see if the health group is saturated before confirming the patient's eligibility on the phone. A health group will be saturated after 250 patients.

If the health group is full, then study staff will let the patient know that she/he is ineligible for the study. Study staff will still need to enter the eligibility survey data into the database and save the data. The database will identify after entering the data that the health group is full. The database will also not allow the patient to be randomized because of the ineligible status based on the full health group. Staff will need to mark the patient as 'ineligible/health status full" under the patient information tab in the database.

If the health group status is not saturated after completing the eligibility survey, patients who are eligible and agree to participate will be asked to come one hour

prior to their scheduled appointment to complete the consent form, receive the intervention booklet or the attention control booklet, and complete a few other research surveys.

4.1.8 **Re-contacting patients**

If Duke staff made 3 attempts to contact the patient at the first provider appointment and the patient was previously unable to be contacted, Duke staff will re-chart the patient's chart when their name is listed on the provider list to check for study eligibility. If the patient's chart information fits study eligibility then the patient is eligible to be mailed a re-contact letter. The patient is instructed in the re-contact letter to contact study staff by phone or by email if the patient is interested in participating in the study. If study staff does not hear from the patient in 10 days then the patient's status is changed in the database to "ineligible for re-contact.

Patients that were originally ineligible for the study from the eligibility survey, but become eligible after re-screen chart review will be contacted via phone by study staff to assess interest in participating in the study. These patients will not be mailed a re-contact letter. Their status in the database is "re-contact call".

When a patient's chart is re-charted and is eligible for the study, Duke staff should go back into the database and set the status to "re-contact letter" for that patient. The process is the same as regular "invitation letters" for sending a re-contact letter. Study staff should have the following materials in front of them before any mailings are conducted: 1) white envelopes 2) access to the EPIC-D database and a printer and 3) a roll of stamps. Study staff will print letters from the EPIC-D database using mail merge. (See Appendix for data entry procedures).

Study staff will print the study invitation letters and labels from the EPIC-D database using mail merge. Staff will place the study invitation letter into a white envelope with the appropriate patient address and place a stamp on the envelope and mail.

Then the RA can go to run the letters and click on "update letter sent". The database will update the data and automatically add a contact that the re-contact letter has been sent. The information will show on the main listing on the "last contact" columns in the database. After, the RA initiates contact again with the patient, the RA needs to change the status in the database to "pending eligibility survey".

Patients that receive the re-contact letter and the decline study participation are marked "declined" in the database.

4.1.9 **Baseline Survey**

This survey may be done over the phone or in person before the patient's visit. (See Appendix for baseline survey). Ideally, study staff should try to complete

the survey on the phone before the patient visit because the window of time on the day of the scheduled appointment for the patient will be short before the doctor's visit.

After finishing the baseline survey questions with the patient over the phone, study staff will remind the patient to come one hour early before the scheduled doctor's visit. Also, staff should tell the patient about the process of enrollment on the day of the appointment. For example, the patient will review and sign the consent form for study participation, review and complete randomization materials, complete a survey before meeting with the doctor, attend appointment with the doctor, and complete another survey after the doctor's appointment.

If the baseline survey was not done on the phone with the patient, then study staff will have the patient review and sign the consent form before completing the baseline survey. After completing the baseline survey, the participant will continue with the rest of the enrollment process: review and complete randomization materials, complete a survey before meeting with the doctor, attend appointment with the doctor, and complete another survey after the doctor's appointment.

4.2 **Day of Patient Appointment**

4.2.1 **Patient No Shows**

If a patient "no shows" for the scheduled appointment, Duke staff will try to reschedule the patient for the study at the next provider's appointment. Staff will change the patient status in the database to "no show for study visit".

4.2.2 **Informed consent**

Duke study staff will bring interested patients into a private room, where they will provide an overview of the study. Staff will describe the study, the compensation, and the time expected of the patient. If study staff was able to contact the patient over the phone and complete the eligibility screener and baseline survey, study staff will move on to administer the study informed consent form (ICF-See Appendix). The study staff will ask the patient whether s/he would like to have the ICF read out loud or read it alone on their own. Depending on the participant's decision about the ICF, staff will then either read the ICF aloud or give the ICF to the patient to read.

After reading the ICF to the patient or after the patient finishes reading the ICF, staff will provide a summary of the form and ask questions to make sure the patient understands what he/she has heard or read. If the patient agrees to participate in the study, the patient will date and sign one copy of the study ICF. Study staff will sign and date the form as a witness. Staff will then make a copy of the form with signatures and date for the participant, make a copy to send to send to Duke medical records, and keep the original for study source documents.

If the person declines to sign the consent, the study staff will ask them for a reason about declining study participation, thank them for their time, and record the reason for declining on the screening eligibility form and the EPIC-D database under patient information status.

After the patient signs the consent form, staff will thank the patient for enrolling and give them a signed copy of the study ICF. Staff will put the ICF along with the other study documents into a study folder. Staff will take all study participant files from the different recruitment sites back to DCRI for storage in a locked room designated for the study.

4.2.3 **Patient Randomization**

Health States

In the EPIC-D database section under the patient information tab, staff will check off the ICF box and provide a date for the informed consent. After saving the page, staff must click on the study participants tab and click on the patient's name at the top of the page. After clicking on the randomization tab, staff can click on the randomization button. Staff must then save the page and pull up the patient's name again under the study participants tab in order to get the patient's health state, study number, and randomization number from the EPIC-D database(See Appendix for data entry instructions).

EPIC-D participants' health states are defined as: best health group, intermediate health group and worst health group. The best health group are those participants who are the youngest with the least comorbidities where previous research studies agreed that screening was likely beneficial for them. Participants in the worst health group who are the oldest participants with the worst comorbidity scores where previous research studies agreed that screening was likely not beneficial for them. Lastly, the intermediate group is for those patients where there was disagreement or where the previous studies could not say whether or not screening would be of net benefit or harm for them. (See table below).

	Co-morbidities Score		
	0	1-3	≥4
70-74	Best	Best	Intermediate
75-79	Best	Intermediate	Worst
80-84	Intermediate	Worst	Worst

4.2.4 **Assigned Randomization Number**

Randomization of Educational Tool

Patients are assigned a randomization number after the patient signs the consent form and completes the baseline survey (if not done over the phone). Participants will be randomized to one of the two study arms in the database. (See appendix for data entry instructions). Participants will have a 50:50 chance of getting assigned to one of the two study arms and either receives the intervention, CRC

Screening brochure and Decision Guide, or the standard of care brochure, Driver 65 Plus and supplement. The decision support intervention is a paper based tool with large font to accommodate visual difficulties with age.

UNC study staff will construct the randomization packets for the Duke staff in increments of 5 randomization numbers per health group. The UNC project manager will deliver to a Duke clinic (TBD) weekly for drop off for use by Duke staff. Duke study staff will take the prefilled constructed envelopes to the practice sites. Duke staff will store the randomization packets at the clinic sites in a locked secure area.

Each study participant will be assigned a random number, (5-6 digits long), which allocates him/her to the control or treatment arm of the study. The random number has been generated by SAS, using a permutation of consecutive blocks. The constructed randomization packets will also have a color coded sticker on the side of the envelope to designate age and gender of the contents in the packets. For example, folders for women age70-74 have a red sticker; 75-79 have a yellow sticker and 80-84 have a gray sticker. Folders for men age 70-74 have a light brown sticker; 75-79 have a lavender sticker; and 80-84 have a dark green sticker.

The study staff will use the participant's health state, age and gender along with the assigned randomization number from the database to determine which packet of information to pull from the pre-stuffed packets to give to the participant. (See Appendix for Intervention and Control materials).

Study staff will instruct the participant to complete the randomization materials in a private room separate from study staff. The study staff will also tell the participant that as part of the study that she cannot answer any study questions about the materials in the envelope because the staff are supposed to be "masked" to which group the participant is randomized to. Rather, the participant should try his/her best to read the materials and answer the questions on the survey supplement. After completing the survey supplement, the participant can keep a copy of the CRC Decision Guide and put all of the other contents back into the study envelope and seal it so study staff is unable to view the materials.

4.2.5 **Post Intervention Survey**

After the participant returns the resealed envelope back to study staff, staff will read and administer the post intervention survey to the participant. (See Appendix for Post Intervention Survey). After completing the post intervention survey, study staff will escort the participant to the waiting room area to check in for the appointment and to wait to be called back by a nurse to the examination room to see the doctor.

4.2.6 **Post Visit Survey**

After the doctor's visit, the participant will return back to the waiting room area where study staff will be waiting for him/her. Study staff will escort the participant to a private area to complete the post visit survey before leaving the clinic (See Appendix for Post Visit Survey). Study staff will read and administer the post visit survey to the participant. Once the survey is completed, the participant will be finished with study surveys for the visit.

5 After the Patient Visit

5.1 Storage of Study Forms

The study staff will place research records in a locked box for transfer from satellite sites to the main site. At the end of the visit, the research records will be transported from the respective satellite sites to the main site securely.

Completed surveys will be kept in the locked offices of the principal investigator's study team at Duke. Twice a month, the UNC project manager will pick up the completed study surveys from Duke study staff. UNC will be responsible for data entering completed surveys and storing them in a locked file cabinet in the UNC principal investigators study research area.

5.1.2 Six Month Follow up

Six months after the participant's visit, they will be contacted by phone by study staff and asked to complete a 10 minute follow up phone questionnaire. (See Appendix for Six Month Follow-up Survey). Study staff should make a minimum of 6 phone call attempts, leaving 3 messages for study participants within a 4 week time period. Staff should not leave more than 1 phone message a week for participants during the follow up period. Study staff will update the participant's status in the database to reflect the contact outcome from the call.

If staff is unable to contact the participant because of a wrong/invalid/ disconnected phone number, study staff will mail a letter to the participant's home address requesting the participant contact study staff for follow up. If study staff does not hear back from the participant after 4 weeks, staff will mark the participant as "lost" in the database.

After 4 weeks of call attempts by study staff, if the participant cannot be reached to complete the 6 month follow-up survey, staff will mail a letter to the participant's home address asking the participant to contact the study team for follow up. Two weeks after mailing the letter to the participant's home address, if study staff doesn't receive a phone call from the participant then staff will mark the participant as "lost" in the database.

5.1.3 Subject Reimbursement

Subjects will be paid at two times throughout their participation in the study. They will receive $25 after their index visit, as well as $25 upon completing their

6 month follow up survey, for a total of $50 for completing all surveys. Study participants will not receive compensation for completing the 12 and 18 month cohort surveys.

Duke study staff has a form that participants are required to complete on their first study visit. The form requires the participants SSN. Duke will not issue a check to participants without them providing their SSN. Participants, who refuse to provide their SSN, can be in the study, but they will not be paid by Duke without providing their SSN.

The signed subject reimbursement form is submitted with an original signature from the participant to the ETR (Employee Travel and Reimbursement) office. An electronic file is submitted to ETR requesting payment. Study staff will then submit a copy of the electronic file along with the original form signed by the participant. ETR generates a check, which is mailed to the participant. This usually takes 4-6 weeks from the time of submission of the payment form. If a participant happens to be a Duke employee, they receive their compensation through direct deposit into the same account as their payroll check.

6 Data Management

Subject confidentiality is held in trust by the participating investigators, their staff, the sponsors, and their agents. The confidentiality extends to the clinical information relating to participating subjects. Participants will be assigned a unique research number (URN). The URN will be used on case report forms. The link between the subjects name and the URN will be kept on a master roster which us kept by the study PI under lock and key.

Full sets of patient data will exist electronically on the DCRI server. Data refers here to all study related information that is specific to an individual patient. These are outlined in the table below. The folder on the DCRI server will always have one copy of the most recent data, which is also backed up several times a day on the server. Hardcopy backups of the surveys should also exist in each participant's file which will be stored at UNC. The Project Manager's (PM) or RA's laptop should not have patient data stored on it.

Data:	Data location:	Task:	When to perform task:	Notes:
Chart abstractions	DCRI server	Upload to server	Daily	
Patient contact information	Hardcopy & database	Enter in database, store at Duke research office	Each contact with patient	Always keep separate from medical data
Loss to follow up control sheets	DM laptop	Upload to UNC	Every week	

Data:	Data location:	Task:	When to perform task:	Notes:
All above patient data	DCRI server	Backup at DCRI	DCRI performs automatically	
Hardcopy eligibility screening forms	Kept on file			Always keep separate from medical data
Other data (Baseline, post intervention, post visit,6 month survey and 12 & 18 month cohort surveys)	Hardcopy at Sheps & Sheps database	Enter in database, store survey data in participant file folder	When new data is available	No identifiers on hardcopies

6.1 **Chart Abstractions**

Chart abstractions are requested for all participants. They will be done manually, using the Chart Review Form (See Chart Review Form in the Appendix). A separate form for each patient must be manually abstracted by consulting Duke EMR. When the chart review is completed, enter data into the database, and then file in the patient's study file. (See Appendix for data entry procedures).

6.2 **Quality Control Measures**

The study coordinator is responsible for overseeing all QC procedures. This will include but is not limited to:

- Contacting all clients who have missed their study appointment.
- Assure that appropriate appointment reminders are being sent out to study patients to remind them of their study visits.
- Maintain complete patient study files with all appropriate study print outs.

Appendix 1: Summary of study events

PROCESS	WHEN	WHO	DESCRIPTION
1.Medical record Chart review	4-6 weeks prior to scheduled PCP appointment	All participants	Use the chart review form to screen for potentially eligible participants.
2. Mail Invitation letter	After chart review	Those pre-screened eligible patients	Patients will be mailed a study invitation letter with a choice to opt out of the study.
3. Eligibility screening	10 days after mailing invitation letter	Those pre-screened eligible patients who are interested in learning more about the study.	The RA will make a minimum of 3 call attempts to reach the patient for study eligibilty before the provider's scheduled appoinment. The RA will ask the patient questions to determine eligibility.
4. Baseline survey	Completed after the eligibility survey	Eligible patients	The RA will answer questions and administer baseline survey over the phone or at the enrollment visit.
5. Introduction	Upon check-in for scheduled PCP appointment	All patients who have agreed to be approached for research, speak English, and are 70-84 years of age	The RA will meet the patient at the front desk and escort to a private room for the study.
6. Enrollment and randomization arm with educational materials	Following screening patient as eligible	Eligible patients	The RA will answer questions and administer ICF. Those who complete ICF will be given a study URN. The RA will randomize the participant and give appropriate educational arm materials to complete.
7. Post intervention Survey	Completed after the educational material before seeing the PCP.	Eligible patients	The RA will administer the post intervention survey after the educational material before seeing the PCP

PROCESS	WHEN	WHO	DESCRIPTION
8. Post vist survey	After the patient's PCP visit, but before leaving the clinic.	Eligible patients	The RA will administer the post visit survey after the patient's PCP visit, but before leaving the clinic.
9. 6 Month follow up Survey	6 months from index visit.	Eligible patients	The RA will call the participant on the phone a minimum of 6 times, leaving at least 3 messages (no more than 1 message a week) within a 4 week time frame 6 months from index visit to complete follow up survey.
10. Quality Control	6 months	All participants	Complete data set collection will be reviewed for quality control by the UNC project manager and PI.

Appendix 2: Study organizational chart

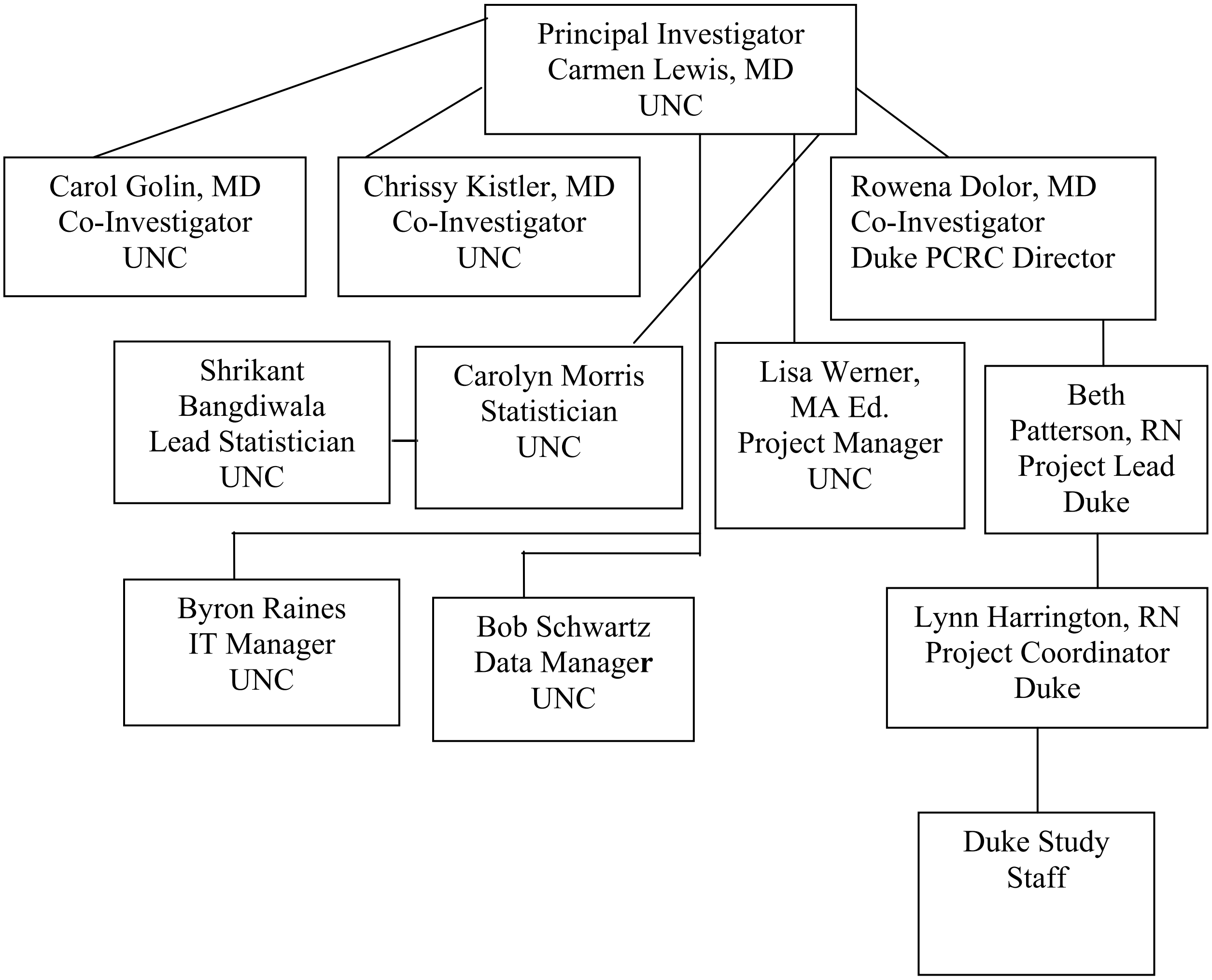

Appendix 3: List of all study forms and additional procedures

1. Chart Review Form
2. Chart Review Procedures for Duke EMR system
3. Invitation Letter
4. Phone Script
5. Data Entry Procedures
6. Eligibility Survey
7. Baseline Survey
8. Randomization Arm-Educational materials
9. Post Intervention Survey
10. Post Visit Survey
11. 6 Month Follow Up
12. Re-contact Letter
13. Re-contact Phone Script
