# Supplementary material for: Effect of a Patient Decision Aid on Preferences for Colorectal Cancer Screening Among Older Adults: A Secondary Analysis of a Randomized Clinical Trial
Source: JAMA Netw Open. 2022 Dec 5;5(12):e2244982. doi: 10.1001/jamanetworkopen.2022.44982 (PMC9855297; doi:10.1001/jamanetworkopen.2022.44982)
Supplement: Supplement 3. — Data Sharing Statement [file jamanetwopen-e2244982-s003.pdf]

# Data Sharing Statement

Dalton. Effect of a Patient Decision Aid on Preferences for Colorectal Cancer Screening Among Older Adults. *JAMA Netw Open*. Published December 05, 2022. doi:10.1001/jamanetworkopen.2022.44982

**Data**
**Data available:** No
